# Psychometric validation of the WHO-5 and WHO-4 well-being index scales for assessing psychological well-being and detecting depression in Japanese school-aged children: a community-based study

**DOI:** 10.3389/fpubh.2025.1662332

**Published:** 2025-12-10

**Authors:** Masaki Adachi, Michio Takahashi, Hiroyuki Mori, Tomoko Nishimura, Makoto Osada, Minami Adachi, Rei Monden, Manabu Wakuta, Kazuhiko Nakamura

**Affiliations:** 1 Department of Psychology, Meiji Gakuin University, Tokyo, Japan; 2 Research Department, Institute of Child Developmental Science Research, Shizuoka, Japan; 3 Department of Neuropsychiatry, Graduate School of Medicine, Hirosaki University, Aomori, Japan; 4 Smart-Aging Research Center, Tohoku University, Sendai, Miyagi, Japan; 5 Faculty of Humanities, Saitama Gakuen University, Saitama, Japan; 6 United Graduate School of Child Development, The University of Osaka, Osaka, Japan; 7 Graduate School of Advanced Science and Engineering, Hiroshima University, Hiroshima, Japan

**Keywords:** WHO-5 well-being index, psychological well-being, cross-cultural validation, confirmatory factor analysis, measurement invariance, depression screening, school-aged children, community sample

## Abstract

**Introduction:**

The 5-item World Health Organization Well-Being Index (WHO-5) is widely used to assess psychological well-being, but its psychometric properties in younger populations, particularly in non-Western settings, remain underexplored. This study aimed to assess the validity and reliability of the Japanese version of the WHO-5 (WHO-5-J) and a culturally adapted four-item version (WHO-4-J), which excludes a culturally sensitive item (“I have felt cheerful and in good spirits”) and uses a simplified four-point Likert scale to minimize cognitive burden among Japanese school-aged children.

**Methods:**

Data were collected from a large community-based sample of 6,983 students aged 10–15 years (Grades 4–9) in Hirosaki City, Japan. We evaluated the factorial validity and internal consistency of the WHO-5-J and WHO-4-J using exploratory and confirmatory factor analyses. Measurement invariance across age and gender groups was examined with multi-group confirmatory factor analyses. Receiver operating characteristic analyses were conducted to determine optimal cutoff scores for detecting psychological distress.

**Results:**

Exploratory and confirmatory factor analyses supported the factorial validity and internal consistency of both the WHO-5-J (α = 0.84–0.88; ω = 0.86–0.91) and WHO-4-J (α = 0.82–0.88), confirming their unidimensional structures. Multi-group confirmatory factor analyses demonstrated full scalar measurement invariance across age and gender groups. Receiver operating characteristic analyses identified optimal cutoff scores with area under the curve values ranging from 0.80 to 0.85, indicating good diagnostic accuracy for psychological distress.

**Discussion:**

Overall, the WHO-4-J demonstrated psychometric properties comparable to those of the WHO-5-J, supporting its practical utility as a culturally appropriate tool for assessing psychological well-being and screening for depression in Japanese youth. These findings underscore the importance of cultural adaptation and developmentally appropriate scaling for accurately assessing psychological well-being in diverse, non-Western child populations.

## 1 Introduction

The 5-item World Health Organization Well-Being Index (WHO-5) is a widely used tool for assessing subjective psychological well-being (1). Developed by the WHO, it comprises five positively framed items that assess mood, interest, and energy levels (2). Each item is rated on a 6-point Likert scale from 0 (“At no time”) to 5 (“All the time”), resulting in a total score ranging from 0 to 25. This score can be converted to a percentage scale (0–100%) for easier interpretation, with higher scores indicating better well-being (3).

Psychological well-being is a crucial aspect of overall quality of life (QoL) and is linked to various life outcomes, including academic success, social adaptation, and long-term mental health (4–6). Reliable measures like the WHO-5 are vital for understanding mental health and guiding effective interventions across diverse populations. The brevity and strong psychometric properties of the WHO-5 make it valuable in clinical and research settings.

In addition to assessing psychological well-being, the WHO-5 has proven to be effective as a screening tool for mental health conditions, particularly depression (7). Previous studies have shown strong negative correlations between WHO-5 scores and established depression measures, such as the Patient Health Questionnaire 9 (PHQ-9) (1, 8, 9), indicating that lower WHO-5 scores can help identify individuals at risk for depressive symptoms.

While the WHO-5 has been extensively validated in adults and clinical populations, its applicability to younger, nonclinical populations—particularly in community settings—remains underexplored (1, 8). Developmental differences in cognition, emotion, and language mean that school-aged children may struggle with abstract or complex well-being items, increasing susceptibility to response bias and reliability concerns (10–13). Consistent with developmental theory and empirical work, age-appropriate, developmentally sensitive tools are needed to capture children’s genuine feelings and to support early mental-health interventions and policy actions from the early school years (14, 15). Moreover, instruments developed or validated in clinical samples may not perform equivalently in community populations, which have broader symptom distributions, lower disorder prevalence, and greater demographic diversity, potentially altering sensitivity and specificity (16). Thus, explicit validation of the WHO-5 in younger, community-based samples is essential to confirm its measurement properties for subjective well-being and to ensure reliable identification of early psychological distress, thereby enabling timely prevention and broad public-health applicability.

Cultural factors shape how WHO-5 items are interpreted and answered, with implications for validity across sociocultural settings (12, 14, 17). Despite its global use, evidence remains concentrated in Europe and North America, with limited coverage of non-Western regions—including East Asia, South Asia, the Middle East, and Africa (18). Within East Asia, for example, studies from China have often focused on university students rather than younger school-age groups, limiting developmental generalizability (19). Cross-cultural work further suggests that expression and reporting of positive affect can vary at the item level (e.g., “I have felt cheerful and in good spirits”), raising potential validity concerns in youth samples (18, 20). These gaps motivate comprehensive research in younger age ranges and diverse contexts within East Asia, including Japan. Because schools increasingly rely on brief well-being screens to enable early identification and stepped support, age-appropriate validation is a prerequisite for safe implementation.

Psychometrically, variability has been reported in factorial structure and cutoffs across cultures and age groups (1, 7). While the WHO-5 is often treated as unidimensional, robust validation requires moving beyond exploratory factor analysis (EFA) to confirmatory factor analysis (CFA) and explicit tests of measurement invariance to avoid biased cross-group comparisons (17, 21–23). A recent cross-national study of 74,071 children and adolescents in 15 European countries found that the WHO-5 did not achieve full measurement invariance across countries, age groups, or genders; scalar non-invariance was especially evident for Item 1 (“cheerful and in good spirits”), motivating a four-item variant (WHO-4) to enhance comparability (20). These signals point not only to potential content-level issues (e.g., Item 1) but also to how response categories function in youth as a plausible driver of scalar non-invariance.

Building on this, developmental questionnaire research shows that six response options can overtax younger respondents, destabilize category thresholds, and thereby undermine scalar invariance (10, 11). Consistent with this rationale, a four-category format may improve interpretability and cross-group comparability in children (18, 20). Accordingly, we formally evaluate WHO-5-J and WHO-4-J head-to-head in Japanese school-aged children, testing whether a four-item content core combined with a four-category response format improves interpretability and scalar comparability beyond mere item reduction. By holding the response format constant (four categories) for both versions, we isolate response-format effects from item-content effects (i.e., the exclusion of Item 1) and can evaluate their respective contributions to measurement invariance and discrimination.

Although the WHO-5 is commonly used as a screening tool for depression, its validation in community-based samples of school-aged children is lacking. Furthermore, the impact of reducing the scale to four items (WHO-4) on its ability to detect depressive symptoms in this population has not been explored. Given the increasing prevalence of mental health challenges among children, exacerbated by the coronavirus disease of 2019 pandemic (24), validating both WHO-5 and WHO-4 in general child populations could be crucial. While the WHO-5 is effective in identifying mild to moderate depressive symptoms, it has limited sensitivity in severe cases, highlighting the need for further refinement (7).

To address these gaps, we evaluated, in a large community sample of Japanese school-aged children, the psychometric validity, reliability, measurement invariance, and screening utility of the WHO-5-J and WHO-4-J under a common four-point Likert response format designed to reduce cognitive burden and enhance response consistency; specifically, we aimed to: (1) conduct a head-to-head psychometric comparison of WHO-5-J and WHO-4-J administered with this shared four-point format; (2) test configural, metric, and scalar invariance across grade (age) and gender; (3) examine convergent validity with established measures of distress and well-being (PHQ-A, KIDSCREEN-10); and (4) determine and compare ROC-based operating characteristics and optimal cut-off scores for detecting psychological distress.

By systematically comparing these two models while holding the response format constant, this study aimed to clarify their comparative suitability for assessing well-being in Japanese school-aged children. The findings could inform school-based mental-health initiatives, guide evidence-based public-health policies, and enhance culturally sensitive mental-health assessments, particularly in non-Western settings.

## 2 Materials and methods

### 2.1 Study design and participants

Data for this study were obtained from the Assessment from Preschool to Puberty—Longitudinal Epidemiological (APPLE) study (25). The APPLE study is a community-based cohort study aimed at clarifying the protective and risk factors and mechanisms of mental health in children and adolescents. Since 2015, data have been collected each September. This study analyzed a cross-sectional wave targeting all elementary and junior high school students in Hirosaki City, Japan. The survey was conducted in collaboration with the Hirosaki City Board of Education and followed established methodologies from previous community-based studies in the same region (e.g., (26, 27)). Hirosaki City is a mid-sized regional municipality in northern Japan (area ≈ 524.20 km^2^; population ≈ 174,171 across ≈ 71,823 households), providing a typical socioeconomic context for public-school populations (25).

The study population included all students in public elementary and junior high schools in Hirosaki City. During the study period, there were 36 elementary schools and 17 junior high schools in the city, with a total enrollment of approximately 11,900 students. The target population consisted of students from Grades 4 through 9, as these students possessed the cognitive and reading abilities necessary to complete the self-reported questionnaires. Private school students, comprising less than 1% of the total student population, were excluded from the study. Coverage of the public-school target population exceeded 99%, supporting representativeness for public-school children in this setting.

Questionnaires were distributed to all eligible students during a designated class period. Teachers explained the study objectives and emphasized the voluntary nature of participation to the students. Additionally, letters outlining the objectives, methods, and ethical considerations were sent to the parents or guardians of all students by email. Parents had the option to withdraw their child from the study by informing the school. Students who were absent on the day of data collection were excluded.

Data was collected in September 2024, with a total of 7,347 questionnaires distributed. Of these, 6,983 questionnaires were completed and returned, resulting in a response rate of 95.0%.

### 2.2 Handling of missing data

Questionnaires with entirely missing responses were excluded (*n* = 102/6,983; 1.46%). Cases with missing values on grouping variables (sex or grade) were retained but excluded only from the relevant grouped analyses; unknown sex/grade was *n* = 8/6,881 (0.12%) among included respondents. For factor models (EFA/CFA and measurement invariance) using ordinal indicators, missing data were handled under the weighted least squares mean- and variance-adjusted (WLSMV) estimator with polychoric correlations, assuming missing at random (MAR). For ROC analyses using PHQ-A as the reference, cases with missing PHQ-A were excluded listwise (*n* = 203/6,881; 2.95%), yielding an effective ROC sample of *n* = 6,678. PHQ-A nonresponse occurred at a low rate (203/6,881; 2.95%) and showed no evidence of concentration by grade [*χ*^2^(5) = 5.710, *p* = 0.336] and only a small sex difference [*χ*^2^(1) = 4.483, *p* = 0.034; male 0.26% vs. female 0.03%]. Taken together, the low overall exclusion rate and minimal subgroup imbalances indicate that missingness is unlikely to meaningfully bias estimates of factor structure, measurement invariance, or ROC-based screening performance, although residual bias cannot be fully excluded.

### 2.3 Ethical considerations

The study was approved by the Committee of Medical Ethics of Hirosaki University Graduate School of Medicine (IRB# 2015–055). Prior to data collection, parents or guardians were provided with written information about the study through schools, with instructions to contact the researchers if they wished to decline participation. Classroom teachers informed students about the study content and their right to decline participation without facing any disadvantages before distributing the questionnaires.

### 2.4 Measures

1. *WHO-5 Well-Being Index (WHO-5-J)*. The WHO-5 is a validated self-report measure designed to assess psychological well-being (1, 3). In Japan, a simplified four-point Likert scale version (S-WHO-5-J) was developed and validated for older adult populations to enhance ease of response while maintaining reliability and validity (28). In this study, we adapted the S-WHO-5-J for school-aged children by adjusting item wording and instructions to be more age-appropriate and understandable for younger participants. A back-translation process was conducted to ensure accuracy and consistency in meaning across translations, and the step-by-step translation workflow (including back translation and native check) is documented in Supplementary Material. The five positively worded items, such as “I have felt cheerful and in good spirits,” assess positive affect, interest, and energy levels over the past 2 weeks. Responses are scored from 0 (“At no time”) to 3 (“All of the time”), yielding a total score ranging from 0 to 15, with higher scores indicating better psychological well-being. Additionally, due to potential cross-cultural variability in interpreting the first item (20), a four-item version (WHO-4) excluding this item was also evaluated.
2. *Patient Health Questionnaire (PHQ)*: The PHQ is a validated self-report tool used to assess depressive symptoms based on the Diagnostic and Statistical Manual of Mental Disorders criteria (29). In this study, the adolescent version (PHQ-A) was used, adapted from the PHQ-9 tailored to the developmental stages of adolescents (30). The PHQ-A comprises nine items that align with the Diagnostic and Statistical Manual of Mental Disorders 5 criteria for major depressive disorder, covering mood, interest, sleep, appetite, energy, self-worth, concentration, psychomotor changes, and suicidal ideation. Each item is rated on a 4-point scale from 0 (“Not at all”) to 3 (“Nearly every day”), resulting in total scores ranging from 0 to 27. A cutoff score of 10 or higher is commonly employed to identify moderate-to-severe depressive symptoms. The psychometric properties of the PHQ-A have been validated in Japanese adolescents, demonstrating its reliability and construct validity in detecting depression (31).
3. *KIDSCREEN-10*: The KIDSCREEN-10 is a validated self-report questionnaire used to assess the general health-related QoL in children and adolescents aged 8 to 18 years (32). This scale is a shortened unidimensional version of the KIDSCREEN questionnaires, comprising 10 items selected from the KIDSCREEN-27 to capture key aspects of physical, psychological, social, and school-related well-being in children. The items cover physical activity, emotional well-being, autonomy, parent–child relationships, peer interactions, and cognitive and learning capacities. Responses are rated on a 5-point Likert scale, indicating frequency (“never” to “always”) or intensity (“not at all” to “extremely”) over the past week. Scores are transformed into T-values with a mean of 50 and a standard deviation of 10, where higher scores indicate better HRQoL. The Japanese version of KIDSCREEN-10 (J-KIDSCREEN-10) has demonstrated acceptable psychometric properties, including internal consistency, test–retest reliability, and concurrent validity in Japanese populations (33).

### 2.5 Data analysis

The data analysis in this study involved several steps to evaluate the psychometric properties and utility of the WHO-5-J. First, descriptive statistics, such as means, standard deviations, and frequency distributions, were computed for the WHO-5, PHQ-A, and KIDSCREEN-10 measures to provide an overview of the data and ensure its suitability for further analysis.

The internal consistency of the WHO-5 and WHO-4 scales among Japanese elementary and junior high school students was assessed in this study. To comprehensively evaluate reliability, both Cronbach’s *α* and McDonald’s *ω* coefficients were utilized. McDonald’s ω is increasingly recommended due to its ability to account for varying factor loadings among items (34, 35). Reliability coefficients of 0.70 or higher were considered acceptable. While Cronbach’s α is commonly used, it may underestimate reliability when factor loadings are not equal and can indicate item redundancy with excessively high alpha values (>0.90) (36). Bayesian estimation methods were employed to calculate McDonald’s ω and Cronbach’s *α*, along with their 95% credible intervals, providing a nuanced understanding of reliability beyond conventional frequentist methods (37). The Bayesian credible intervals enhanced interpretability by explicitly quantifying uncertainty around reliability estimates, strengthening the robustness of the findings.

EFA and CFA are commonly employed together to assess the construct validity of psychological scales. Typically, EFA is initially conducted to identify the underlying factor structure without imposing a preconceived theoretical model. Subsequently, CFA is performed to assess the fit of the hypothesized model derived from the EFA results (38). It is recommended to split the sample into two independent subsets for EFA and CFA to prevent overfitting, validate the factor structure identified by EFA independently, and enhance the validation process’s robustness (38). While there is no universally prescribed ratio for dividing samples, it is generally recommended to allocate a larger portion to EFA for stable factor extraction and a smaller portion to CFA for validation (39). In the present study, we followed a 2:1 ratio of participants for EFA to CFA, aligning with these recommendations. Additionally, ensuring comparability between subsets in terms of key demographic variables like gender and grade level is crucial to reduce biases and enhance result generalizability (38). Therefore, we randomly divided the analytic sample (*N* = 6,873) into independent EFA and CFA subsets with a 2:1 allocation (EFA: *n* = 4,811; CFA: *n* = 2,062), implemented in Python (Jupyter; pandas, numpy, scikit-learn StratifiedShuffleSplit, seed = 2025). Stratification was performed jointly by sex (1 = male, 2 = female) and school grade (4–9) to preserve marginal distributions. Post-split balance was excellent. For sex, the male proportion was 47.37% (EFA) vs. 47.37% (CFA); the difference in proportions was Δ = 0.000 percentage points [95% CI (−1.80, 1.80)]. A Pearson’s chi-square test indicated no association between subset and sex, *χ*^2^(1) = 0.000, *p* = 0.999, with a negligible effect size (*φ* = 0.000). For grade (4–9), distributions were also equivalent, *χ*^2^(5) = 0.007, *p* = 0.999, Cramér’s *V* = 0.001. This methodological approach adheres to established psychometric best practices, indicating that with clearly defined factor structures and adequate sample sizes, population-level factor structures can be accurately reproduced even without a perfect model fit (38). Thus, the approach adopted in this study enhances the reliability and validity of the identified factor structures.

Because item responses are ordinal Likert categories, all factor models were estimated from polychoric correlations using the weighted least squares mean and variance adjusted (WLSMV) estimator with a probit link, which does not assume multivariate normality and is robust to non-normality. We screened item-level distributions (skewness and kurtosis) and found values within acceptable ranges; total scores were approximately normal, supporting the use of parametric summaries. Within this framework, exploratory factor analyses applied a Geomin oblique rotation to allow correlated factors (40). Factor retention was determined based on various criteria, including eigenvalues greater than 1 (41), scree plot inspection (42), and model fit indices. Solutions with one to five factors were considered, but convergence was only achieved for one- and two-factor solutions.

CFA was conducted on the second subset using the same WLSMV estimator. A unidimensional model was specified for each version: all five items for the WHO-5 and all four items for the WHO-4 were set to load onto a single latent factor. The factor variance was fixed at 1, and the mean was set to 0 for model identification.

The model fit for both EFA and CFA was assessed using the Comparative Fit Index (CFI), Tucker-Lewis Index (TLI), Root Mean Square Error of Approximation (RMSEA), and Standardized Root Mean Square Residual (SRMR). Acceptable fit criteria were defined as CFI/TLI ≥ 0.90 and RMSEA ≤ 0.08, while good fit was defined as CFI/TLI ≥ 0.95 and RMSEA ≤ 0.06; SRMR values ≤0.10 and ≤ 0.08 were considered acceptable and good, respectively (43). RMSEA values slightly above 0.06 were considered acceptable due to the low number of items in both scales, consistent with prior recommendations for low degrees of freedom (44, 45).

Following the CFA, subgroup differences were examined through multi-group confirmatory factor analysis using the entire sample (*n* = 6,873) to assess measurement invariance across gender and age groups. The analysis proceeded sequentially, starting with configural invariance to ensure consistency in factor structure across groups, followed by metric invariance to assess equivalence in factor loadings, and scalar invariance to ascertain if item intercepts remained invariant across groups. Model fit was assessed based on established criteria: CFI > 0.95, TLI > 0.95, and RMSEA < 0.06 (43). However, it is important to consider potential bias in RMSEA values for models with low degrees of freedom, particularly with fewer than 10 indicators (45), as is the case with both the WHO-5 and WHO-4 scales. Moreover, Hu and Bentler (43) indicated that RMSEA values close to 0.06 are acceptable, while values ≤0.08 are widely recognized as acceptable. Therefore, RMSEA values ranging approximately from 0.06 to 0.08 were considered indicative of an acceptable model fit for the present study (22, 23, 45). Upon establishing configural invariance, metric invariance and scalar invariance were tested. Model comparisons were not conducted using chi-square difference tests due to the test’s sensitivity to large sample sizes. Instead, differences in CFI and TLI values ≤0.01 and differences in RMSEA ≤0.015 were considered indicative of measurement invariance.

To assess concurrent validity, Pearson’s correlation coefficients were computed to investigate the associations between the WHO-5-J and two other measures, the PHQ-A and KIDSCREEN-10. These correlations offer insights into the alignment of the WHO-5-J with established measures of depressive symptoms and health-related QoL.

To determine the optimal cutoff scores for both the WHO-5-J and WHO-4-J, ROC curve analyses were conducted. The PHQ-A was used as the reference measure for identifying psychological distress, with predefined cutoff scores of ≥10 for moderate depression, ≥15 for moderately severe depression, and ≥20 for severe depression. The discriminative ability of each scale was assessed by calculating the area under the ROC curve (AUC), with values ≥0.90 indicating excellent discriminative accuracy, 0.80–0.89 representing good accuracy, 0.70–0.79 indicating fair accuracy, and values below 0.70 suggesting poor accuracy (46). AUCs were reported with 95% confidence intervals computed by the DeLong method, and paired DeLong tests were used to compare AUCs between WHO-4-J and WHO-5-J. Various diagnostic accuracy indices, including sensitivity (the proportion correctly identifying true positive cases), specificity (the proportion correctly identifying true negative cases), the Youden’s index (defined as sensitivity + specificity-1, which indicates the optimal balance between sensitivity and specificity (47)), positive likelihood ratios (LR+), and negative likelihood ratios (LR−), were analyzed to determine optimal cutoff scores. LR + values greater than 5 indicate strong diagnostic utility, values between 2 and 5 suggest moderate utility, and LR− values less than 0.2 indicate a low risk of false negatives (48, 49). The overall accuracy of correctly classified cases was assessed, with optimal cutoff scores determined by maximizing the Youden’s index to ensure balanced diagnostic precision (47). In community-based primary screening contexts, minimizing false negatives is crucial, so alternative thresholds emphasizing higher sensitivity with acceptable trade-offs in specificity were also considered. The ROC analyses, incorporating AUC values, sensitivity, specificity, Youden’s index, likelihood ratios, and overall classification accuracy, provided comprehensive evidence for selecting optimal cutoff points and assessing the discriminative accuracy of the WHO-5-J and WHO-4-J across varying levels of depressive symptoms.

Descriptive statistics, reliability estimates, and correlation analyses were conducted using JASP (Version 0.19.3). EFA and CFA, including multi-group CFA, were performed using Mplus (Version 8.8). ROC analyses were conducted using IBM Statistical Package for the Social Sciences Statistics (Version 28.0.1.0).

## 3 Results

### 3.1 Descriptive statistics

Table 1 presents the descriptive statistics for each item in the WHO-5 questionnaire, along with the total scores for both WHO-5 and WHO-4. All items demonstrated acceptable levels of skewness and kurtosis, indicating their suitability for subsequent psychometric analyses. The total scores for WHO-4 and WHO-5 had distributions close to normal, with skewness values of −0.716 for WHO-5 and −0.709 for WHO-4, and kurtosis values of −0.014 for WHO-5 and −0.048 for WHO-4, supporting their use in parametric analyses (50).

**Table 1:** Descriptive statistics, item-total correlations, and internal consistency estimates for the WHO-5 and WHO-4 Items.

Item/scale	*N*	Mean	SD	sk	Ku	*r*_it_	*ω*_iid_	*α*_iid_
WHO1	6,867	2.331	0.705	−0.742	−0.026	0.812	0.813	0.818
WHO2	6,861	2.275	0.749	−0.728	−0.119	0.730	0.835	0.839
WHO3	6,839	2.336	0.773	−0.895	−0.015	0.767	0.824	0.829
WHO4	6,835	2.035	0.890	−0.472	−0.763	0.635	0.866	0.868
WHO5	6,872	2.452	0.727	−1.124	0.492	0.719	0.837	0.842
WHO-5 total score	6,804	11.439	3.116	−0.716	−0.014			
WHO-4 total score	6,807	9.106	2.531	−0.709	−0.048			

sk, skewness; ku, kurtosis; *r*_it_, corrected item-total correlations; *α*_iid_, Cronbach’s alpha, if item deleted, ω_iid_, Cronbach’s alpha, if item deleted.

Tables 2, 3 present the frequency distributions of total scores for WHO-5 and WHO-4, respectively. Both tables revealed negatively skewed distributions, with a majority of students reporting high levels of psychological well-being. Around 57% of participants scored 12 or higher on WHO-5 (out of a maximum of 15), while approximately 63% scored 10 or higher on WHO-4 (out of a maximum of 12), indicating generally favorable psychological states in the sample. The amount of missing data was minimal (2.56% for WHO-5, 2.52% for WHO-4), further confirming the reliability of the data for subsequent analyses.

**Table 2:** Frequency distribution of WHO-5 total scores among Japanese school-aged children (*n* = 6,983).

Observed value	Frequency	Probability (%)	Valid percentage	Cumulative percentage
0	20	0.29	0.29	0.29
1	10	0.14	0.15	0.44
2	17	0.24	0.25	0.69
3	32	0.46	0.47	1.16
4	65	0.93	0.96	2.12
5	181	2.59	2.66	4.78
6	208	2.98	3.06	7.83
7	266	3.81	3.91	11.74
8	381	5.46	5.60	17.34
9	540	7.73	7.94	25.28
10	865	12.39	12.71	37.99
11	612	8.76	8.99	46.99
12	622	8.91	9.14	56.13
13	665	9.52	9.77	65.90
14	785	11.24	11.54	77.44
15	1,535	21.98	22.56	100.00
Missing value	179	2.56		
Total	6,983	100	100	

WHO-5, 5-item World Health Organization Well-Being Index; Observed value, WHO-5 total score; Frequency, number of respondents; Probability (%), percentage of total sample including missing values; Valid Percentage, percentage of valid (non-missing) responses; Cumulative percentage, cumulative percentage of valid responses. Missing values accounted for 2.56% of the total sample.

**Table 3:** Frequency distribution of WHO-4 total scores among Japanese school-aged children (*n* = 6,983).

Observed value	Frequency	Probability (%)	Valid percentage	Cumulative percentage
0	21	0.30	0.31	0.31
1	19	0.27	0.28	0.59
2	35	0.50	0.51	1.10
3	70	1.00	1.03	2.13
4	221	3.16	3.25	5.38
5	285	4.08	4.19	9.56
6	454	6.50	6.67	16.23
7	603	8.64	8.86	25.09
8	964	13.80	14.16	39.25
9	772	11.06	11.34	50.59
10	830	11.89	12.19	62.79
11	927	13.28	13.62	76.41
12	1,606	23.00	23.59	100.00
Missing value	176	2.52		
Total	6,983	100	100	

WHO-4, 4-item World Health Organization Well-Being Index; Observed value, WHO-4 total score; Frequency, number of respondents; Probability (%), percentage of total sample including missing values; Valid percentage, percentage of valid (non-missing) responses; Cumulative percentage, cumulative percentage of valid responses. Missing values accounted for 2.52% of the total sample.

### 3.2 Reliability and internal consistency

The WHO-5 scale demonstrated strong internal consistency, with a McDonald’s *ω* of 0.864 (95% confidence interval [CI]: 0.859–0.869) and a Cronbach’s *α* of 0.867 (95% CI: 0.862–0.872). Similarly, the WHO-4 scale had a McDonald’s ω of 0.813 (95% CI: 0.805–0.820) and a Cronbach’s α of 0.818 (95% CI: 0.810–0.824). Both scales exceeded the recommended threshold of 0.80 for reliability coefficients, indicating robust internal consistency (36). These reliability statistics are detailed in Table 4. Analysis of the impact of removing individual items from the WHO-5 scale revealed minor fluctuations in the reliability coefficients (α range: 0.818–0.868). Deleting the WHO4 item resulted in the highest reliability coefficient (*α* = 0.868), suggesting a slightly weaker contribution compared to the other items. However, the marginal impact of item deletion indicates that each item contributes meaningfully to the overall internal consistency of the scale, supporting the structural validity observed in previous studies (1, 8). Detailed results of the item analysis are presented in Table 1.

**Table 4:** Internal consistency and concurrent validity of WHO-5 and WHO-4 with PHQ-A and KIDSCREEN-10.

		WHO-5	WHO-4
Internal consistency	Cronbach’s α	0.867 (95% CI: 0.862–0.872)	0.818 (95% CI: 0.810–0.824)
	McDonald’s ω	0.864 (95% CI: 0.859–0.869)	0.813 (95% CI: 0.805–0.820)
Pearson’s correlation coefficient			
WHO-4	*r*	0.990	–
	*p*-value	< 0.001	–
	95%CI	0.990 ~ 0.991	–
PHQ-A	*r*	−0.603	−0.598
	*p*-value	< 0.001	< 0.001
	95%CI	−0.618 ~ −0.588	−0.613 ~ −0.583
Kidscreen10	*r*	0.757	0.741
	*p*-value	< 0.001	< 0.001
	95%CI	0.747 ~ 0.767	0.730 ~ 0.752

WHO-5, 5-item World Health Organization Well-Being Index; WHO-4, modified four-item version of WHO-5; PHQ-A, Patient Health Questionnaire for Adolescents; KIDSCREEN-10, 10-item measure of Health-Related Quality of Life for children and adolescents; CI, confidence interval; *r* = Pearson’s correlation coefficient; all correlation coefficients were statistically significant at *p* < 0.001.

### 3.3 Exploratory factor analysis

The eigenvalues showed a clear “elbow,” supporting a dominant first factor (WHO-5: 3.763, 0.486, 0.333, 0.274, 0.143; WHO-4: 2.938, 0.464, 0.333, 0.265). A one-factor EFA provided acceptable-to-good fit for both scales [WHO-5: *χ*^2^(5) = 144.685, RMSEA = 0.076 (90% CI 0.066–0.087), CFI = 0.997, TLI = 0.994, SRMR = 0.023; WHO-4: *χ*^2^(2) = 69.614, RMSEA = 0.084 (0.068–0.101), CFI = 0.996, TLI = 0.989, SRMR = 0.023]. Consistent with prior recommendations, model evaluation relied on multiple indices (RMSEA with 90% CIs, CFI, TLI, SRMR) (43, 51); the slightly elevated RMSEA for WHO-4 is expected in short, low-df models and does not contradict the overall one-factor solution (45).

All factor loadings were significant (*p* < 0.001). Loadings ranged 0.701–0.945 for WHO-5 (highest on Item 1 = 0.945) and 0.724–0.861 for WHO-4, indicating a clear unidimensional structure in both versions. Because WHO-4 excludes the strongest-loading item, its maximum loading and explained variance are modestly lower, yet the scale retains adequate measurement precision. Full item-level estimates are provided in Table 5.

**Table 5:** Exploratory and confirmatory factor analysis of the WHO-5 and WHO-4 items among Japanese school-aged children.

WHO1–WHO5		Exploratory factor analysis				Confirmatory factor analysis			
		WHO-5		WHO-4		WHO-5		WHO-4	
Item		Factor loading	Residual variance	Factor loading	Residual variance	Factor loading	Residual variance	Factor loading	Residual variance
WHO1	I have felt cheerful and in good spirits.	0.945	0.107	(Removed)	(Removed)	0.944	0.109	(Removed)	(Removed)
WHO2	I have felt calm and relaxed.	0.812	0.341	0.815	0.335	0.809	0.346	0.814	0.337
WHO3	I have felt active and vigorous.	0.879	0.228	0.861	0.259	0.874	0.237	0.854	0.271
WHO4	I woke up feeling fresh and rested.	0.701	0.508	0.724	0.476	0.702	0.507	0.727	0.471
WHO5	My daily life has been filled with things that interest me.	0.821	0.326	0.821	0.326	0.825	0.320	0.822	0.324
N		4,811		4,811		2062		2062	
Model fit									
Chi-square		144.685		69.614		79.19		25.1	
Degrees of freedom		5		2		5		2	
Relative chi-square (*χ*^2^/df)		28.94		34.81		15.84		12.55	
*p*-value		<0.001		<0.001		<0.001		<0.001	
RMSEA		0.076		0.084		0.079		0.081	
RMSEA 90% CI		[0.066, 0.087]		[0.068, 0.101]		[0.063, 0.096]		[0.057, 0.108]	
CFI		0.997		0.996		0.997		0.996	
TLI		0.994		0.989		0.993		0.989	
SRMR		0.023		0.023		0.014		0.012	

WHO-5, 5-item World Health Organization Well-Being Index; WHO-4, modified four-item version excluding item WHO1 (“I have felt cheerful and in good spirits”); RMSEA, Root Mean Square Error of Approximation; CI, confidence interval; CFI, Comparative Fit Index; TLI, Tucker–Lewis Index; SRMR, Standardized Root Mean Square Residual. All factor loadings and residual variances were statistically significant at *p* < 0.001.

### 3.4 Confirmatory factor analysis

The model fit indices indicated a good overall fit for both WHO-5 and WHO-4. For WHO-5, the chi-square statistic was 79.19 (df = 5, *p* < 0.001), RMSEA = 0.079 (90% CI: 0.063–0.096), CFI = 0.997, TLI = 0.993, and SRMR = 0.014. For WHO-4, the chi-square statistic was 25.1 (df = 2, *p* < 0.001), RMSEA = 0.081 (90% CI: 0.068–0.096), CFI = 0.996, TLI = 0.989, and SRMR = 0.012. Detailed model fit indices and factor loadings are presented in Table 5.

Although the RMSEA values for the WHO-4 model slightly exceed the conventional cutoff of 0.08 for an acceptable fit (38), previous research has indicated that RMSEA may be biased in models with low degrees of freedom (45), particularly when the number of indicators is fewer than 10, as in this study. Given the limited number of items in both WHO-5 and WHO-4, the slightly elevated RMSEA may not necessarily indicate a poor fit. Furthermore, the CFI and TLI values surpassed the widely accepted threshold of 0.95 for good model fit (43), and the SRMR values were well below the recommended cutoff of 0.08 (51), further supporting the adequacy of both models.

All standardized factor loadings were significant (*p* < 0.001), supporting a clear unidimensional structure in both versions. Consistent with the EFA, Item 1 showed the strongest loading in WHO-5; its exclusion accounts for the slightly lower maximum loading in WHO-4, while the short form retains adequate measurement precision. These findings suggest that while the WHO-4 scale remains a valid measure of well-being, the WHO-5 scale may offer a more robust representation of the construct due to the inclusion of WHO-1.

The *R*-square values were generally high for both scales, indicating strong measurement properties. WHO-5: *R*-square ranged 0.492 (Item 4)–0.893 (Item 1); residual variances were 0.107 (Item 1), 0.341 (Item 2), 0.228 (Item 3), 0.508 (Item 4), 0.326 (Item 5). WHO-4: R-square ranged 0.524 (Item 4)–0.741 (Item 3); residual variances were 0.335 (Item 2), 0.259 (Item 3), 0.476 (Item 4), 0.326 (Item 5). The higher R-square in WHO-5—especially for Item 1 (0.893)—indicates that omitting this strongest indicator in WHO-4 modestly reduces the maximum explained variance; nevertheless, WHO-4 maintains adequate explanatory power and remains a viable short form.

### 3.5 Measurement invariance of the WHO-5 and WHO-4 across grade groups

A stepwise measurement invariance analysis was conducted to determine if the WHO-5 and WHO-4 Well-Being Indexes function equivalently across elementary and junior high school students. The analysis assessed configural, metric, and scalar invariance models to evaluate the consistency of well-being measurements across grade groups. Results are summarized in Table 6.

**Table 6:** Measurement invariance testing of WHO-5 and WHO-4 across grade levels in Japanese school-aged children.

Grade	Models	Model fit indices							Model Comparison					
		*χ* ^2^	df	*χ*^2^/df	CFI	TLI	RMSEA	SRMR	ΔCFI	ΔTLI	ΔRMSEA	*χ* ^2^	df	*p*-value
WHO-5	Configural	155.634	10	15.56	0.986	0.972	0.065	0.019	—	—	—	—	—	—
	Metric	198.283	14	14.16	0.982	0.974	0.062	0.044	−0.004	−0.002	0.003	38.892	4	*p* < 0.001
	Scalar	294.788	18	16.38	0.973	0.970	0.067	0.057	−0.009	0.004	−0.005	108.776	4	*p* < 0.001
WHO-4	Configural	82.581	4	20.65	0.987	0.961	0.076	0.017	—			—	—	
	Metric	115.203	7	16.46	0.982	0.969	0.067	0.040	−0.005	−0.008	0.009	28.973	3	*p* < 0.001
	Scalar	224.466	10	22.45	0.966	0.960	0.078	0.057	−0.016	0.009	−0.011	109.263	3	*p* < 0.001

WHO-5, 5-item World Health Organization Well-Being Index; WHO-4, modified four-item version excluding item WHO1; *χ*^2^, chi-square statistic; df, degrees of freedom; CFI, Comparative Fit Index; TLI, Tucker–Lewis Index; RMSEA, Root Mean Square Error of Approximation; SRMR, Standardized Root Mean Square Residual; Δ, Change between nested models.

The configural invariance model, which examined the overall factor structure of the WHO-5 and WHO-4 without imposing equality constraints, demonstrated an adequate fit. The WHO-5 model yielded a chi-square value of 155.634 (df = 10), RMSEA of 0.065 (90% CI: 0.056–0.074), CFI of 0.986, TLI of 0.972, and SRMR of 0.019, indicating that the single-factor model applied to both elementary and junior high school students. The WHO-4 model also demonstrated an acceptable fit with *χ*^2^(4) = 82.581, RMSEA = 0.076 (90% CI: 0.062–0.090), CFI = 0.987, TLI = 0.961, and SRMR = 0.017. These results suggest that well-being measurement was consistent across grade groups for both scales. However, the slightly higher RMSEA and lower TLI for the WHO-4 model may be due to the exclusion of WHO-1.

After establishing configural invariance, metric invariance was tested by constraining factor loadings to be equal across groups. For WHO-5, the chi-square difference test showed a significant difference between the metric and configural models [Δ*χ*^2^(4) = 38.892, *p* < 0.001], but the overall model fit remained strong, with *χ*^2^(14) = 198.283, RMSEA = 0.062, CFI = 0.982, TLI = 0.974, and SRMR = 0.044. The change in CFI (ΔCFI = −0.004) was below the commonly used threshold of −0.01, supporting metric invariance. Similarly, for WHO-4, a significant difference was found between the metric and configural models [Δχ^2^(3) = 28.973, *p* < 0.001], with fit indices within acceptable limits [*χ*^2^(7) = 115.203, RMSEA = 0.067, CFI = 0.982, TLI = 0.969, SRMR = 0.040]. The change in CFI (ΔCFI = −0.005) further supports metric invariance, indicating that the relationships between the latent well-being construct and individual items were equivalent across grade groups for both WHO-5 and WHO-4.

Scalar invariance was assessed by constraining item intercepts to be equal across groups. For the WHO-5 scale, the chi-square difference test comparing the scalar and metric models showed a significant difference [Δ*χ*^2^(4) = 108.776, *p* < 0.001]. However, the overall model fit remained acceptable, with *χ*^2^(18) = 294.788, RMSEA = 0.067, CFI = 0.973, TLI = 0.970, and SRMR = 0.057. The change in CFI (ΔCFI = −0.009) was within the −0.01 threshold, supporting full scalar invariance for meaningful comparisons of latent well-being across grade groups. In contrast, for the WHO-4 scale, the scalar model showed a significant decrease in fit compared to the metric model, with Δ*χ*^2^(3) = 109.263, *p* < 0.001, and a CFI decline of −0.016, exceeding the recommended threshold. These results indicate that the WHO-4 scale does not fully meet the criteria for scalar invariance, indicating potential differences in response tendencies between elementary and junior high school students.

Overall, the results of measurement invariance analysis indicate that the WHO-5 scale offers a more stable and generalizable assessment of well-being across different grade levels, meeting the criteria for full scalar invariance. In contrast, the WHO-4 scale only demonstrated partial scalar invariance, implying that score differences may not solely reflect variations in latent well-being but could be influenced by variations in item response patterns among grade groups. This implies that adjustments for partial scalar invariance may be needed to ensure the comparability of latent means when using the WHO-4 scale. The slightly higher RMSEA values observed in all models for the WHO-4 scale indicate that the removal of WHO-1 may have impacted the scale’s structural integrity. Therefore, the WHO-5 scale appears to be a more robust and reliable measure for assessing well-being in elementary and junior high school students.

### 3.6 Measurement invariance of WHO-5 and WHO-4 across gender groups

To assess the comparability of the WHO-5 and WHO-4 Well-Being Index across gender groups, a series of measurement invariance tests were conducted. The analysis followed a hierarchical approach, assessing the configural, metric, and scalar invariance models sequentially. The results are summarized in Table 7.

**Table 7:** Measurement invariance testing of WHO-5 and WHO-4 across gender groups in Japanese school-aged children.

Gender	Models	Model fit indices							Model comparison					
		*χ* ^2^	df	*χ*^2^/df	CFI	TLI	RMSEA	SRMR	ΔCFI	ΔTLI	ΔRMSEA	*χ* ^2^	df	*p*-value
WHO-5	Configural	155.634	10	15.56	0.987	0.974	0.063	0.019	—	—	—	—	—	—
	Metric	168.129	14	13.01	0.985	0.978	0.057	0.031	−0.002	−0.004	0.006	17.57	4	*p* < 0.001
	Scalar	224.299	18	12.46	0.980	0.977	0.058	0.041	−0.005	0.001	−0.001	58.601	4	*p* < 0.001
WHO-4	Configural	75.568	4	18.89	0.988	0.964	0.072	0.017	—			—	—	—
	Metric	96.385	7	13.77	0.985	0.974	0.061	0.031	−0.003	−0.01	0.011	16.651	3	*p* < 0.001
	Scalar	152.183	10	15.22	0.976	0.971	0.065	0.043	−0.004	0.003	−0.004	59.355	3	*p* < 0.001

WHO-5, 5-item World Health Organization Well-Being Index; WHO-4, modified four-item version excluding item WHO1; *χ*^2^, chi-square statistic; df, degrees of freedom; CFI, Comparative Fit Index; TLI, Tucker–Lewis Index; RMSEA, Root Mean Square Error of Approximation; SRMR, Standardized Root Mean Square Residual; Δ, Change between nested models.

The configural invariance model demonstrated an adequate fit for both the WHO-5 and WHO-4. The WHO-5 model showed a chi-square value of 155.634 (df = 10), RMSEA = 0.063 (90% CI: 0.054–0.072), CFI = 0.987, TLI = 0.974, and SRMR = 0.019, confirming that the single-factor structure applied to both males and females. Similarly, the WHO-4 model exhibited acceptable fit indices, with *χ*^2^(4) = 75.568, RMSEA = 0.072 (90% CI: 0.059–0.087), CFI = 0.988, TLI = 0.964, and SRMR = 0.017. These findings indicate that the latent well-being construct is similarly structured across gender groups in both versions of the scale.

To examine whether the factor loadings were equivalent across gender groups, metric invariance was examined by constraining the factor loadings to be equal. For the WHO-5 model, the chi-square difference test comparing the metric and configural models yielded a significant result [Δ*χ*^2^(4) = 17.57, *p* < 0.001]. However, the overall model fit remained strong, with *χ*^2^(14) = 168.129, RMSEA = 0.057 (90% CI: 0.049–0.065), CFI = 0.985, TLI = 0.978, and SRMR = 0.031. The change in CFI (ΔCFI = −0.002) was below the commonly accepted threshold of −0.01, supporting metric invariance. Similarly, for the WHO-4 model, metric invariance was supported despite a significant chi-square difference [Δ*χ*^2^(3) = 16.651, *p* = 0.0008], as the model fit indices remained within an acceptable range *χ*^2^(7) = 96.385, RMSEA = 0.061 (90% CI: 0.051–0.072), CFI = 0.985, TLI = 0.974, SRMR = 0.031, with a ΔCFI = −0.003. These results indicate that the relationships between the latent well-being factor and individual items were equivalent across gender groups in both the WHO-5 and WHO-4 models.

Finally, scalar invariance was tested by constraining item intercepts to be equal across gender groups. For the WHO-5 scale, the chi-square difference test comparing the scalar and metric models was statistically significant [Δ*χ*^2^(4) = 58.601, *p* < 0.001]. However, the overall model fit remained acceptable, with χ^2^(18) = 224.299, RMSEA = 0.058 (90% CI: 0.051–0.065), CFI = 0.980, TLI = 0.977, and SRMR = 0.041. The change in CFI (ΔCFI = −0.005) did not exceed the −0.01 threshold, supporting full scalar invariance. Similarly, for the WHO-4 scale, the scalar invariance model also showed a significant chi-square difference [Δ*χ*^2^(3) = 59.355, *p* < 0.001], but the changes in CFI (ΔCFI = −0.004) remained within an acceptable range. The final model fit for the WHO-4 scale was *χ*^2^(10) = 152.183, RMSEA = 0.065 (90% CI: 0.056–0.074), CFI = 0.976, TLI = 0.971, and SRMR = 0.043. These findings indicate that both the WHO-5 and WHO-4 scales meet the criteria for full scalar invariance.

Overall, these findings suggest that both the WHO-5 and WHO-4 scales demonstrate full scalar invariance across gender groups, enabling direct comparisons of latent well-being scores between males and females. The stability of the CFI and the minor impact on RMSEA values further support the robustness of these measures. The achievement of full scalar invariance by both scales suggests that any observed mean differences in well-being scores between genders reflect true differences in the latent construct rather than measurement artifacts. These findings underscore the suitability of both the WHO-5 and WHO-4 scales for gender-based comparisons of well-being, providing strong empirical evidence for their measurement equivalence.

### 3.7 Convergent validity

To assess convergent validity, Pearson’s correlation coefficients were computed between the WHO-5, WHO-4, and relevant constructs. The WHO-5 scale exhibited a strong correlation with the WHO-4 scale (*r* = 0.990), a negative correlation with depressive symptoms (PHQ-A; *r* = −0.603), and a positive correlation with subjective well-being (KIDSCREEN-10; *r* = 0.757). Similarly, the WHO-4 scale was negatively correlated with PHQ-A (*r* = −0.598) and positively correlated with KIDSCREEN-10 (*r* = 0.741). Based on Cohen’s criteria (52), these coefficients reflect large effect sizes, supporting the convergent validity of both the WHO-5 and WHO-4 scales. Detailed correlation coefficients and confidence intervals are presented in Table 4.

### 3.8 Receiver operating characteristic curve analysis

**Figure 1:**
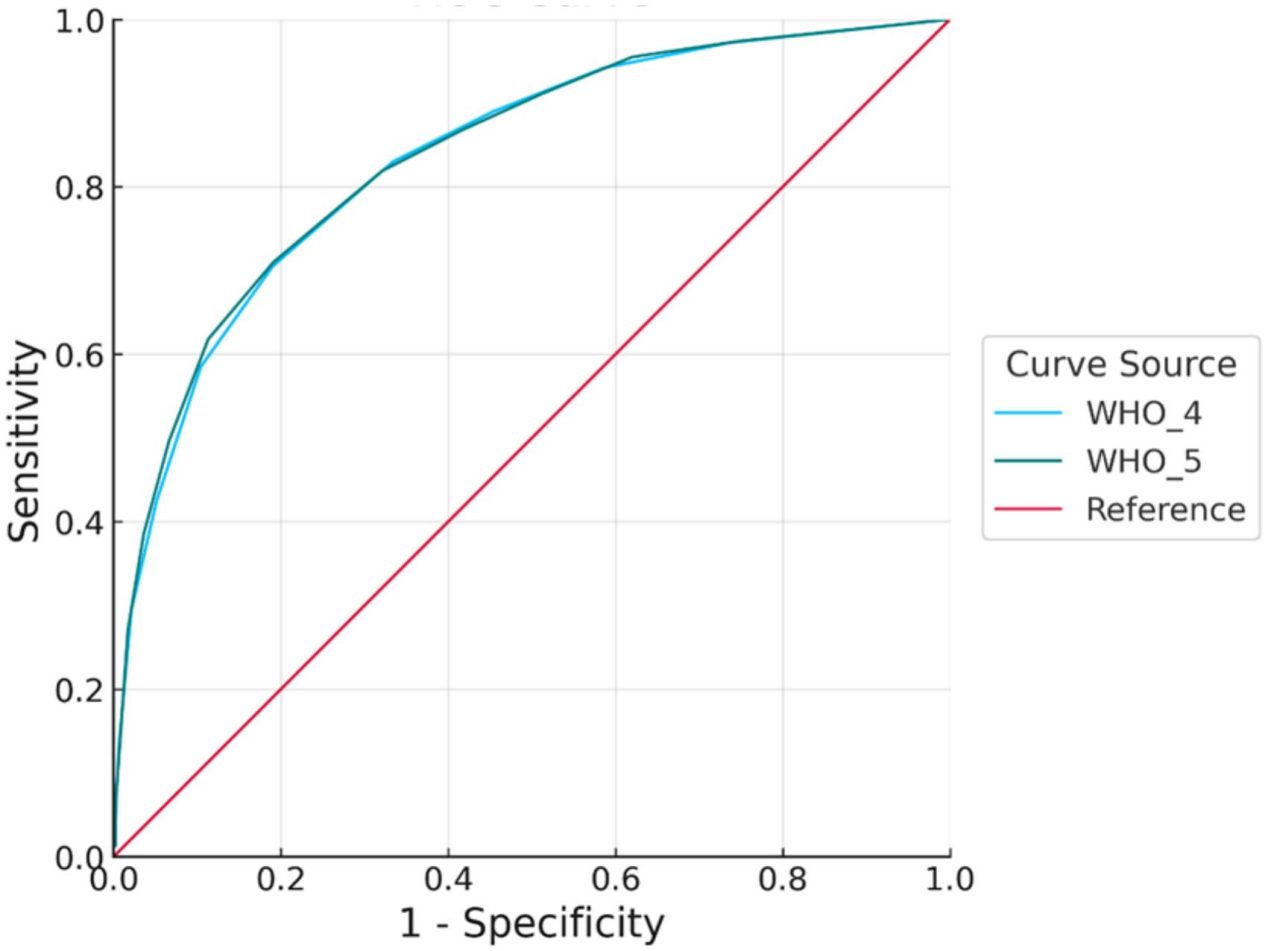
ROC curve for moderate depression. Receiver operating characteristic (ROC) curves for the WHO-4 and WHO-5 well-being indices in detecting moderate depression. The *x*-axis represents the false positive rate (1 − specificity), and the *y*-axis represents the true positive rate (sensitivity). The WHO-5 showed marginally better discriminative performance than the WHO-4, though the difference was minimal. ROC, receiver operating characteristic; WHO-4, 4-item version of the World Health Organization-Five Well-Being Index; WHO-5, 5-item version of the World Health Organization-Five Well-Being Index; AUC, area under the curve.

**Figure 2:**
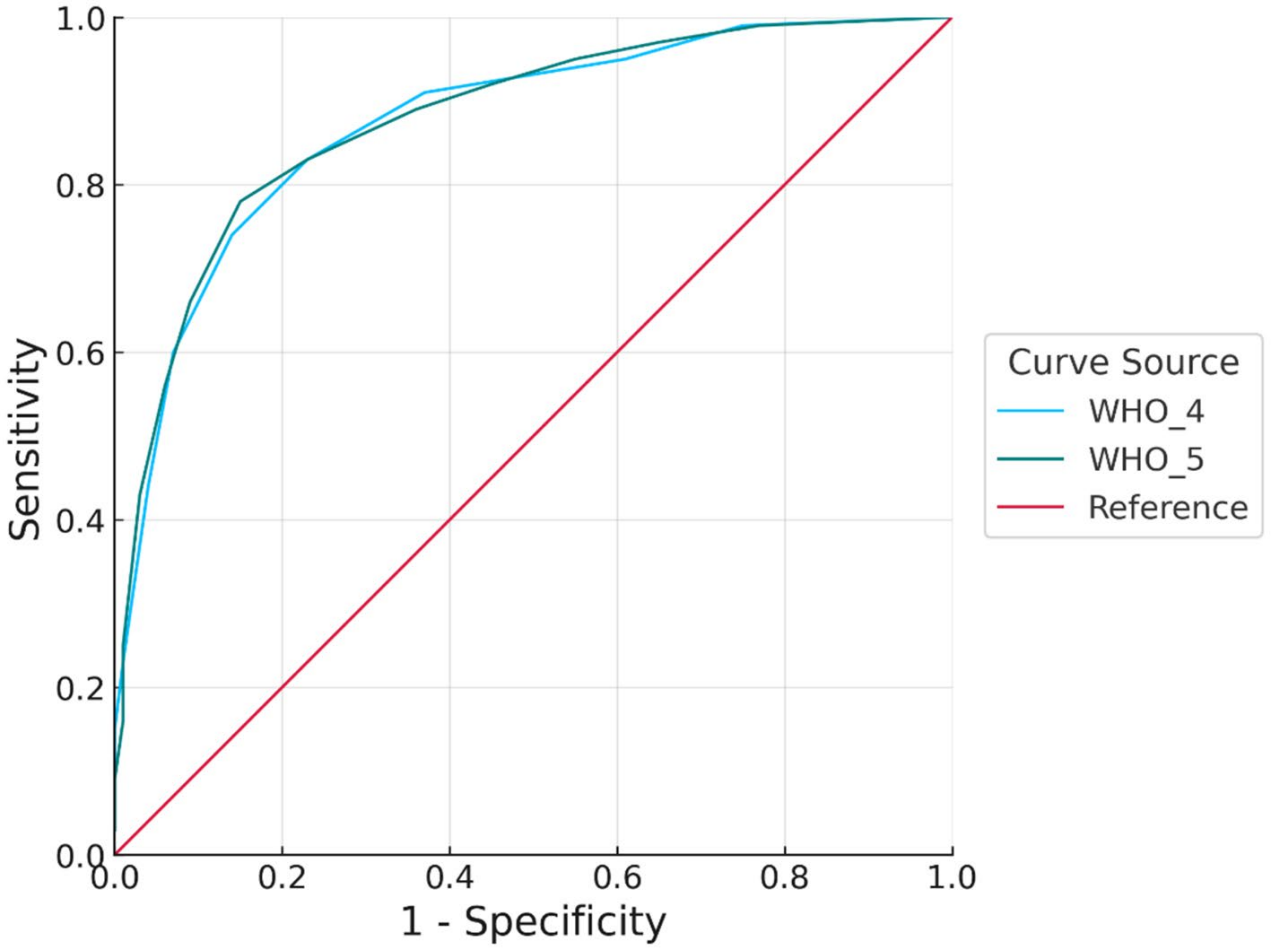
ROC curve for moderately severe depression. Both WHO-4 and WHO-5 demonstrated high discriminative ability, with ROC curves approaching the upper left corner. WHO-5 showed slightly better sensitivity at comparable levels of specificity, suggesting a marginal advantage in identifying moderately severe depressive symptoms. ROC, receiver operating characteristic; WHO-4, 4-item version of the World Health Organization-Five Well-Being Index; WHO-5, 5-item version of the World Health Organization-Five Well-Being Index; AUC, area under the curve.

**Figure 3:**
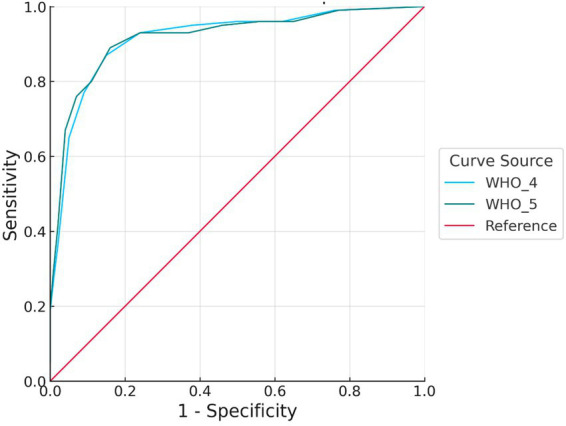
ROC curve for severe depression. Both WHO-4 and WHO-5 demonstrated high accuracy, particularly at lower false positive rates. WHO-5 exhibited slightly higher sensitivity than WHO-4 across several cut points, suggesting its potential advantage in screening for severe depressive symptoms. ROC, receiver operating characteristic; WHO-4, 4-item version of the World Health Organization-Five Well-Being Index; WHO-5, 5-item version of the World Health Organization-Five Well-Being Index; AUC, area under the curve.

The ROC analysis results, as summarized in Table 8 and illustrated in Figures 1–3, indicated that both the WHO-4 and WHO-5 scales demonstrated strong discriminative accuracy in identifying depression across various severity levels (moderate, moderately severe, and severe). For moderate depression, the AUC was 0.84 (95% CI: 0.82–0.85) for WHO-4 and 0.84 (95% CI: 0.82–0.85) for WHO-5; the paired DeLong test indicated no significant difference (ΔAUC = 0.00, 95% CI: −0.01–0.01, *p* > 0.05). This is consistent with the classic interpretation that substantial overlap of 95% confidence intervals rarely implies a statistically significant difference (53).

**Table 8:** Diagnostic accuracy of WHO-4 and WHO-5 for detecting different levels of depression severity in Japanese school-aged children.

Dependent variable (depression severity)	Predictor	AUC	SE	Asymptotic *p*-value	95% CI		*N* (%)		
					Lower	Upper	Positive	Negative	Missing
Moderate depression	WHO-4	0.84	0.008	<0.001	0.82	0.85	772 (11.6)	5,906 (88.4)	305
	WHO-5	0.84	0.008	<0.001	0.82	0.85			
Moderately severe depression	WHO-4	0.87	0.012	<0.001	0.85	0.90	259 (3.9)	6,419 (96.1)	
	WHO-5	0.88	0.012	<0.001	0.85	0.90			
Severe depression	WHO-4	0.92	0.018	<0.001	0.88	0.95	75 (1.1)	6,603 (98.9%)	
	WHO-5	0.92	0.019	<0.001	0.88	0.96			

WHO-5, 5-item World Health Organization Well-Being Index; WHO-4, modified four-item version excluding item WHO1; AUC, area under the curve; SE, standard error; CI, confidence interval; *N* (%), number and percentage of participants classified according to depression severity using PHQ-A cutoff scores (≥10 points for moderate depression, ≥15 points for moderately severe depression, and ≥20 points for severe depression). All analyses were statistically significant at *p* < 0.001. Higher area under the curve values indicate better diagnostic accuracy.

A similar trend was observed for moderately severe depression, with an AUC of 0.87 (95% CI: 0.85–0.90) for WHO-4 and 0.88 (95% CI: 0.85–0.90) for WHO-5; the paired DeLong test indicated no significant difference (ΔAUC = 0.01, 95% CI: −0.00–0.02, *p* > 0.05). Although WHO-5 showed slightly higher accuracy, the substantial overlap of the confidence intervals and the very small ΔAUC indicate that the difference is not statistically or clinically meaningful.

In the case of severe depression, both scales demonstrated very high discriminative performance, with an AUC of 0.92 (95% CI: 0.88–0.95) for WHO-4 and an AUC of 0.92 (95% CI: 0.88–0.96) for WHO-5; the paired DeLong test indicated no significant difference (ΔAUC = 0.00, 95% CI: −0.02–0.02, *p* > 0.05). At this severity level, the accuracy of the two scales was nearly identical, with no meaningful differences.

Overall, the WHO-5 scale consistently exhibited slightly higher discriminative accuracy than the WHO-4 scale across all levels of depression severity, although these differences were not statistically significant. Both scales demonstrate practical effectiveness in identifying depression. The WHO-4 scale could be sufficiently useful as a general screening tool for broadly detecting depressive symptoms; however, the WHO-5 scale may be preferred in situations where specificity and minimizing false positives are crucial. In clinical settings, the choice between the WHO-4 and WHO-5 scales should be based on the specific screening objectives and the required diagnostic accuracy (46).

To address potential heterogeneity in measurement precision by developmental stage, we additionally conducted age-stratified ROC analyses (Elementary: Grades 4–6; Junior-high: Grades 7–9). For both WHO-4 and WHO-5, discrimination remained good to excellent within each stratum, and the 95% confidence intervals of the AUCs overlapped across age groups. On this basis, we proceeded with pooled analyses for subsequent models. Stratum-specific AUCs and case/control counts are provided in the Supplementary Tables.

### 3.9 Optimal cutoff points for the WHO-4 and WHO-5 scales by depression severity

Optimal cutoff points for detecting depression at different severity levels (moderate, moderately severe, and severe) using WHO-4 and WHO-5 scales were determined based on sensitivity, specificity, Youden’s index, LR+, LR−, and overall classification accuracy (46, 47, 53). The detailed results are summarized in Table 9.

**Table 9:** Sensitivity, specificity, likelihood ratios, and positive predictive values of WHO-4 and WHO-5 at various cut-off points for detecting moderate, moderately severe, and severe depression among Japanese school-aged Children.

Scale	Moderate depression							Moderately severe depression						Severe depression						PPV estimation	
	CP	SE	SP	YI	CC	LR+	LR−	SE	SP	YI	CC	LR+	LR−	SE	SP	YI	CC	LR+	LR−	12-month	Cumulative
WHO-4	≤1	0.01	1.00	0.01	0.51	7.00	0.99	0.03	1.00	0.02	0.51	13.50	0.97	0.05	1.00	0.05	0.53	26.50	0.95	16.5%	28.9%
	≤2	0.04	1.00	0.03	0.52	18.00	0.97	0.07	1.00	0.07	0.54	24.33	0.93	0.19	1.00	0.18	0.59	46.75	0.82	33.7%	51.2%
	≤3	0.07	1.00	0.07	0.54	24.33	0.93	0.15	1.00	0.14	0.57	29.40	0.86	0.28	0.99	0.27	0.64	35.00	0.73	40.8%	58.6%
	≤4	0.13	0.99	0.12	0.56	21.67	0.88	0.23	0.99	0.22	0.61	19.00	0.78	0.36	0.98	0.34	0.67	21.18	0.65	38.0%	55.8%
	≤5	0.29	0.98	0.27	0.64	14.00	0.72	0.44	0.96	0.40	0.70	11.89	0.58	0.65	0.95	0.61	0.80	14.20	0.36	28.4%	44.9%
	≤6	0.42	0.95	0.37	0.69	8.31	0.61	0.60	0.93	0.52	0.76	8.04	0.44	0.77	0.91	0.69	0.84	8.99	0.25	19.0%	32.6%
	≤7	0.59	0.90	0.48	0.74	5.63	0.46	0.74	0.86	0.60	0.80	5.38	0.30	0.87	0.85	0.71	0.86	5.70	0.16	13.7%	24.7%
	≤8	0.71	0.81	0.52	0.76	3.71	0.36	0.83	0.77	0.60	0.80	3.65	0.22	0.93	0.76	0.69	0.85	3.86	0.09	9.5%	17.8%
	≤9	0.83	0.67	0.50	0.75	2.49	0.26	0.91	0.63	0.54	0.77	2.45	0.15	0.95	0.62	0.56	0.78	2.46	0.09	6.6%	12.6%
	≤10	0.89	0.55	0.44	0.72	1.96	0.20	0.93	0.51	0.45	0.72	1.92	0.13	0.96	0.50	0.46	0.73	1.92	0.08	5.3%	10.2%
	≤11	0.94	0.41	0.36	0.68	1.61	0.14	0.95	0.39	0.34	0.67	1.55	0.12	0.96	0.38	0.34	0.67	1.54	0.11	4.3%	8.6%
	≤12	0.97	0.26	0.24	0.62	1.32	0.11	0.99	0.25	0.23	0.62	1.30	0.06	0.99	0.24	0.23	0.61	1.30	0.05	3.6%	7.1%
WHO-5	≤1	0.01	1.00	0.01	0.51	6.50	0.99	0.03	1.00	0.02	0.51	13.50	0.97	0.05	1.00	0.05	0.53	26.50	0.95	15.5%	27.4%
	≤2	0.03	1.00	0.02	0.51	12.50	0.98	0.05	1.00	0.04	0.52	15.33	0.96	0.11	1.00	0.10	0.55	35.67	0.90	26.1%	42.1%
	≤3	0.04	1.00	0.04	0.52	21.50	0.96	0.09	1.00	0.09	0.55	31.00	0.91	0.20	1.00	0.20	0.60	40.00	0.80	37.8%	55.6%
	≤4	0.08	1.00	0.07	0.54	25.00	0.93	0.16	0.99	0.15	0.58	26.33	0.85	0.31	0.99	0.30	0.65	38.37	0.70	41.4%	59.3%
	≤5	0.13	0.99	0.12	0.56	18.57	0.88	0.25	0.99	0.23	0.62	20.58	0.76	0.41	0.98	0.40	0.70	25.81	0.60	34.4%	51.9%
	≤6	0.27	0.98	0.25	0.63	15.94	0.74	0.43	0.97	0.39	0.70	13.71	0.59	0.67	0.96	0.63	0.81	16.67	0.35	31.1%	48.1%
	≤7	0.39	0.96	0.35	0.68	10.72	0.64	0.56	0.94	0.50	0.75	9.75	0.47	0.76	0.93	0.69	0.85	11.01	0.26	23.3%	38.4%
	≤8	0.50	0.93	0.43	0.72	7.52	0.54	0.66	0.91	0.57	0.78	7.02	0.38	0.80	0.89	0.69	0.85	7.41	0.22	17.5%	30.4%
	≤9	0.62	0.89	0.50	0.75	5.47	0.43	0.78	0.85	0.63	0.81	5.28	0.26	0.89	0.84	0.73	0.87	5.48	0.13	13.4%	24.1%
	≤10	0.71	0.81	0.52	0.76	3.72	0.36	0.83	0.77	0.60	0.80	3.64	0.22	0.93	0.76	0.69	0.84	3.82	0.09	9.5%	17.8%
	≤11	0.82	0.68	0.50	0.75	2.55	0.27	0.89	0.64	0.53	0.77	2.49	0.17	0.93	0.63	0.56	0.78	2.51	0.11	6.7%	12.9%
	≤12	0.87	0.58	0.45	0.73	2.08	0.23	0.92	0.55	0.47	0.73	2.04	0.15	0.95	0.54	0.48	0.74	2.05	0.10	5.6%	10.8%
	≤13	0.91	0.49	0.40	0.70	1.77	0.18	0.95	0.45	0.40	0.70	1.74	0.12	0.96	0.44	0.40	0.70	1.73	0.09	4.8%	9.4%
	≤14	0.96	0.38	0.33	0.67	1.54	0.12	0.97	0.35	0.33	0.66	1.51	0.08	0.96	0.35	0.30	0.65	1.47	0.12	4.2%	8.2%
	≤15	0.97	0.25	0.23	0.61	1.30	0.10	0.99	0.23	0.22	0.61	1.29	0.06	0.99	0.23	0.21	0.61	1.28	0.06	3.6%	7.0%

CP, Cut-off point; SE, Sensitivity; SP, Specificity; YI, Youden’s Index; CC, Correctly classified; LR+, Positive likelihood ratio; LR−, Negative likelihood ratio; PPV, Positive predictive value. Sensitivity indicates the proportion of true positive cases identified correctly, while specificity represents the proportion of true negative cases identified correctly. Youden’s Index (YI = Sensitivity + Specificity − 1) reflects the optimal balance between sensitivity and specificity. The Correctly classified (CC) metric shows the overall accuracy, representing the proportion of participants accurately identified as positive or negative. Positive likelihood ratio (LR+) indicates how much more likely individuals with depression are to have a positive screening result compared to those without depression; conversely, Negative likelihood ratio (LR−) indicates how much less likely individuals with depression are to have a negative screening result compared to those without depression. Higher LR+ values and lower LR− values indicate greater diagnostic accuracy. PPVs were estimated based on 12-month prevalence (2.75%) and cumulative prevalence (5.5%), demonstrating the proportion of screening-positive individuals who are likely to have depression, noting that PPV varies depending on the underlying prevalence.

For moderate depression, the recommended clinical screening thresholds were set at ≤8 points for WHO-4 (sensitivity = 0.71, specificity = 0.81, Youden’s index = 0.52, correctly classified = 76%, LR+ = 3.71, LR− = 0.36) and ≤10 points for WHO-5 (sensitivity = 0.71, specificity = 0.81, Youden’s index = 0.52, correctly classified = 76%, LR+ = 3.72, LR − = 0.36). Both thresholds demonstrated balanced diagnostic utility suitable for clinical and general screening contexts (48, 49).

For moderately severe depression, the optimal thresholds were slightly higher, at ≤7 points for WHO-4 (sensitivity = 0.74, specificity = 0.86, Youden’s index = 0.60, correctly classified = 80%, LR+ = 5.38, LR− = 0.30) and ≤9 points for WHO-5 (sensitivity = 0.78, specificity = 0.85, Youden’s index = 0.63, correctly classified = 81%, LR+ = 5.28, LR− = 0.26). These thresholds provided improved diagnostic accuracy, featuring strong likelihood ratios and acceptable false-negative rates (53).

For severe depression, the identified optimal thresholds were also ≤7 points for WHO-4 (sensitivity = 0.87, specificity = 0.85, Youden’s index = 0.71, correctly classified = 86%, LR+ = 5.70, LR− = 0.16) and ≤9 points for WHO-5 (sensitivity = 0.89, specificity = 0.84, Youden’s index = 0.73, correctly classified = 87%, LR+ = 5.48, LR− = 0.13). These thresholds yielded excellent diagnostic accuracy with minimal false negatives, making them particularly suitable for identifying severe cases requiring immediate intervention (46, 53).

In practical scenarios like community-based primary screening, where high sensitivity is prioritized to minimize the risk of missed cases (false negatives), the recommended cutoff points were adjusted slightly upward (toward higher scores) to ensure greater sensitivity, accepting minor reductions in specificity. Specifically, for moderate depression, the recommended thresholds were adjusted to ≤9 points for WHO-4 (sensitivity = 0.83, specificity = 0.67) and ≤11 points for WHO-5 (sensitivity = 0.82, specificity = 0.68). For moderately severe depression, thresholds of ≤8 points for WHO-4 (sensitivity = 0.83, specificity = 0.77) and ≤10 points for WHO-5 (sensitivity = 0.83, specificity = 0.77) were recommended. In the context of severe depression, community-based screening thresholds were similarly set at ≤8 points for WHO-4 (sensitivity = 0.93, specificity = 0.76) and ≤10 points for WHO-5 (sensitivity = 0.93, specificity = 0.76), providing excellent sensitivity for early identification in broader community settings (48, 49).

Based on a comprehensive analysis of various severity levels and considering sensitivity, specificity, and overall accuracy, it is recommended to establish unified optimal cutoff points for practical clinical and community-based use at ≤8 points for WHO-4 and ≤10 points for WHO-5. These standardized thresholds demonstrate consistent and robust diagnostic performance while effectively balancing the trade-offs between false positives and false negatives (46). In community settings where early detection is crucial to minimize missed cases, slightly higher unified thresholds are suggested: ≤9 points for WHO-4 and ≤11 points for WHO-5. These adjusted thresholds improve the chances of early identification, enabling timely preventive interventions (54–57).

## 4 Discussion

The present study significantly advances the literature by validating the WHO-5 Well-Being Index and its abbreviated version (WHO-4) among a large, community-based sample of Japanese school-aged children. Through rigorous psychometric analyses, we confirmed the reliability of both scales, factorial validity, measurement invariance across gender and age groups, convergent validity, and optimal screening thresholds for psychological distress. This study addresses important gaps regarding the suitability of the WHO-5 scale for younger, nonclinical populations in East Asia, particularly in educational settings where early identification of psychological distress is crucial. By using a simplified four-point Likert scale to reduce cognitive load, our approach innovatively expands the practical utility and accessibility of the WHO-5 scale among younger respondents, which is beneficial for large-scale school screenings (10, 11).

The close correspondence between *α* and *ω* indicates limited heterogeneity in item loadings and thus strong structural coherence (58). The obtained reliability estimates are consistent with prior validations of the WHO-5 and comparable work in diverse populations, supporting generalizability and cross-cultural stability (1, 8, 18, 20). The use of Bayesian estimation further enhanced the interpretability of the reliability coefficients by providing precise credible intervals, supporting the robustness of the findings (37). Item removal analysis indicated that deleting any single item from the WHO-5 scale did not meaningfully improve reliability, suggesting that each item contributes effectively to the overall internal consistency of the scale. Although item WHO4 showed a slightly lower corrected item-total correlation, its removal did not substantially affect reliability, supporting the retention of all items in practical applications.

Factorial analyses confirmed a clear unidimensional structure, consistent with existing literature that identifies the WHO-5 scale as measuring a single construct of subjective psychological well-being (1, 3). Both EFA and CFA consistently supported this single-factor structure, further reinforcing the established factorial validity of the WHO-5 scale in diverse populations (19, 20). Measurement invariance analyses revealed full scalar invariance across gender for both WHO-5 and WHO-4, indicating that gender-based comparisons of well-being are unbiased. However, while WHO-5 demonstrated full scalar invariance across grade groups, WHO-4 only showed partial scalar invariance. Full scalar invariance is critical to ensure that gender- and age-based comparisons of latent means accurately reflect true differences in well-being without bias (17, 22, 23). The partial scalar invariance observed in WHO-4 suggests that caution and potential adjustments are necessary when interpreting cross-grade comparisons, highlighting the superiority of WHO-5 for broader developmental applications.

A significant methodological innovation in our study was the modification of the standard 6-point Likert scale to a simplified 4-point scale to reduce cognitive burden in children, as younger respondents may struggle with extensive response options (10, 11). Although this scaling change prevented direct numeric comparisons with previous studies, it did not compromise discriminative metrics such as sensitivity, specificity, and the AUC. Indeed, our observed AUCs in the range of 0.85–0.90 were consistent with prior research using the standard WHO-5 format (7, 59), demonstrating that the overall diagnostic power remained intact despite the scale modification.

Our ROC analysis confirmed that both WHO-5 and WHO-4 have good accuracy in detecting depression across varying severity levels. This study is the first to formally evaluate WHO-4’s screening performance in a large community-based sample of school-aged children, suggesting its utility as a practical short-form tool in educational settings. Optimal cutoff scores were identified by considering sensitivity-specificity trade-offs, clinical utility, and predictive values influenced by the prevalence of depression. Positive predictive value (PPV) strongly depends on contextual prevalence rates (60–62), and we incorporated global epidemiological prevalence estimates to interpret the screening results. Specifically, the 12-month prevalence for adolescents aged 10–15 years was estimated at 2.75%, based on WHO reports (63) and meta-analytic findings (64). The cumulative prevalence was set at 5.5%, reflecting longitudinal data indicating a lifetime prevalence of approximately 11% among adolescents aged 13–18 (65).

As shown in Table 9, sensitivity, specificity, and PPV varied significantly based on the chosen thresholds for the WHO-4 and WHO-5 scales. Thresholds with the highest specificity (e.g., WHO-4 ≤ 3, WHO-5 ≤ 6) had very low sensitivity, posing a risk of missing cases. Conversely, thresholds identified as statistically optimal using Youden’s index (WHO-4 ≤ 8, WHO-5 ≤ 10) offered a balanced diagnostic accuracy, making them suitable for initial screening. However, it is crucial to consider practical and clinical factors alongside statistical optimization. Indeed, Brehaut et al. (54) emphasized the importance of thresholds that account for local prevalence rates, resource availability, and the impact of missed diagnoses.

Therefore, slightly adjusted thresholds (WHO-4 ≤ 9, WHO-5 ≤ 11) were recommended to improve sensitivity and minimize false negatives, prioritizing early detection and timely intervention in community-based settings like schools. This adjustment aligns closely with public health principles outlined in Wilson and Jungner’s screening criteria (66, 67). While these adjusted thresholds inevitably reduce PPV through an increased false-positive rate, this trade-off is justified by the clinical and ethical imperative to identify vulnerable children early, provided that there are adequate psychological support services, trained professionals, and effective referral systems in place.

Indeed, the effectiveness of screening programs depends on selected thresholds and the availability of follow-up resources and intervention capacity (68–70). Inadequate infrastructure can result in unintended consequences such as increased anxiety, stigma from mental health labeling, or delayed intervention due to overwhelmed resources (66, 70). This highlights the need for flexible screening strategies that consider local resources, prevalence rates, and ethical considerations regarding the psychological impact on those screened. For instance, in resource-limited school settings, a more specific threshold may be preferred to avoid overlooking existing services, while in well-equipped settings with robust follow-up capabilities, higher sensitivity thresholds are needed for early detection.

Ultimately, the selection of optimal screening thresholds should be guided by clear objectives, careful assessment of local prevalence, available resources, and consideration of potential psychological and ethical implications. Previous WHO-5 studies have often used a single cutoff point irrespective of severity. In contrast, our identification of severity-specific thresholds offers tailored guidance based on severity, a novel contribution that enriches the existing literature on mental health screening. While we report recommended operating cut-offs, threshold selection should remain adaptable to local prevalence and follow-up capacity; in this spirit, we tabulated the full range of candidate cut-offs with their operating characteristics so that districts can calibrate decisions to their resources. Therefore, our findings offer clear and practical recommendations for clinical practice and community-based mental-health policies, effectively bridging statistical precision and clinical utility in school-based depression screening.

These results underscore the inherent limitations of single-stage screenings and emphasize the necessity of context-sensitive, multitiered approaches. When implementing school-wide screenings, it is crucial to select thresholds in light of local resources and follow-up capacity. In resource-limited settings, more conservative (higher-specificity) thresholds can increase PPV and concentrate scarce follow-up on higher-risk cases; conversely, in well-resourced settings, more inclusive (higher-sensitivity) thresholds may facilitate early intervention (66, 68).

Finally, our findings have significant implications for school-wide mental health initiatives, supporting the routine integration of the WHO-5 scale—and potentially the WHO-4 scale as a streamlined alternative—for early depression screening. At a policy level, our results justify district-wide screening frameworks that assign complementary roles to the two versions: WHO-5 for unbiased cross-grade monitoring (given its full scalar invariance) and WHO-4 as a streamlined option when maximizing coverage is prioritized in routine workflows. Many adolescents experiencing psychological distress go unnoticed in school settings, and using brief, positively framed well-being questionnaires like the WHO-5 scale can enhance honest reporting, reduce stigma, and enable early intervention (59, 71). Specifically, the positive framing of the WHO-5 items may reduce the stigma associated with overt mental health questions, enhancing the acceptability and accuracy of student responses in school-wide mental health programs (1, 3, 20). Effective school-based screening programs require robust follow-up infrastructure to translate screening results into tangible mental-health benefits (69, 70). Framing the instruments in this way facilitates routine integration into school health policy (universal screening with stepped follow-up) and enables population-level dashboards to track grade-level trends and guide resource allocation.

Despite the significant strengths of our study, it is important to acknowledge several limitations. First, the cross-sectional design of our study limited our ability to assess the predictive validity and longitudinal stability of the WHO-5 and WHO-4 measures. Future longitudinal studies are necessary to determine their predictive capabilities for mental health outcomes over time. Second, external validity is bounded: although the sampling frame—restricted to public elementary and junior-high schools—supports internal validity, generalizability to private-school settings, to areas with different socioeconomic profiles, or to non-Japanese contexts remains uncertain, particularly because individual-level SES was not collected. Future studies should recruit multi-site samples across prefectures and school types, incorporate individual or area-level SES indicators, and undertake cross-national replications to establish broader applicability.

Additionally, criterion validity in this study was based solely on a self-report reference (PHQ-A). To address this limitation, future research should include brief, blinded subsample clinical validation using standardized diagnostic interviews to confirm AUCs (with 95% CIs) and sensitivity/specificity at prespecified cut points, and to examine potential age-related heterogeneity under a clinical reference. Lastly, while our study identified optimal cutoffs balancing sensitivity and specificity, the appropriateness of these thresholds depends on contextual factors such as local prevalence rates, resource availability, and specific screening goals. Therefore, practitioners should interpret and adjust these cutoffs flexibly, considering their specific operational contexts and available resources (54, 55, 57).

In conclusion, our study supports the psychometric robustness and practical utility of the WHO-5 and WHO-4 scales as screening tools for psychological well-being in Japanese school-aged children. Addressing the identified limitations through longitudinal designs, clinical diagnostic validation, and geographical replications in future research will enhance their applicability and clinical utility.

## 5 Conclusion

The present study offers comprehensive validation evidence supporting the WHO-5 and WHO-4 scales as effective, reliable, and culturally appropriate screening tools for identifying psychological distress among Japanese school-aged children. Our findings establish optimal cutoff thresholds that balance sensitivity and specificity, enabling evidence-based decisions tailored to specific contextual needs and resource capacities within school-based mental health programs. Practitioners are encouraged to adapt these thresholds thoughtfully, considering their screening objectives, local prevalence rates, and available follow-up infrastructure. Future research should further validate these tools through longitudinal studies, clinical diagnostic comparisons, and replication in diverse populations and settings to ensure their ongoing relevance and effectiveness in global mental health screening efforts.

## Data Availability

The dataset analyzed in this study is part of an ongoing prospective cohort study and is therefore not publicly available at present. Researchers who wish to use the data may contact the corresponding author with a reasonable request; data sharing will be considered in line with the study’s ethical approvals and data-sharing agreements.

## References

[1] Topp CW Østergaard SD Søndergaard S Bech P. The WHO-5 well-being index: a systematic review of the literature. Psychother Psychosom. (2015) 84:167–76. doi: 10.1159/000376585, 25831962

[2] World Health Organization. 1998. Wellbeing measures in primary health care/the DepCare project: Report on a WHO meeting: Stockholm, Sweden, 12–13 February 1998. Regional office for Europe. Geneva: World Health Organization. Regional Office for Europe. Available online at: https://iris.who.int/handle/10665/349766

[3] Bech P Olsen LR Kjoller M Rasmussen NK. Measuring well-being rather than the absence of distress symptoms: a comparison of the SF-36 mental health subscale and the WHO-five well-being scale. Int J Methods Psychiatr Res. (2003) 12:85–91. doi: 10.1002/mpr.145, 12830302 PMC6878541

[4] Diener E Suh EM Lucas RE Smith HL. Subjective well-being: three decades of progress. Psychol Bull. (1999) 125:276–302. doi: 10.1037/0033-2909.125.2.276

[5] Huppert FA. Psychological well-being: evidence regarding its causes and consequences. Appl Psychol Health Well Being. (2009) 1:137–64. doi: 10.1111/j.1758-0854.2009.01008.x

[6] Lyubomirsky S King L Diener E. The benefits of frequent positive affect: does happiness lead to success? Psychol Bull. (2005) 131:803–55. doi: 10.1037/0033-2909.131.6.803, 16351326

[7] Krieger T Zimmermann J Huffziger S Ubl B Diener C Kuehner C . Measuring depression with a well-being index: further evidence for the validity of the WHO well-being index (WHO-5) as a measure of the severity of depression. J Affect Disord. (2014) 156:240–4. doi: 10.1016/j.jad.2013.12.015, 24412323

[8] Awata S Bech P Koizumi Y Seki T Kuriyama S Hozawa A . Validity and utility of the Japanese version of the WHO-five well-being index in the context of detecting suicidal ideation in elderly community residents. Int Psychogeriatr. (2007) 19:77–88. doi: 10.1017/S1041610206004212, 16970832

[9] Ghazisaeedi M Mahmoodi H Arpaci I Mehrdar S Barzegari S. Validity, reliability, and optimal cut-off scores of the WHO-5, PHQ-9, and PHQ-2 to screen depression among university students in Iran. Int J Ment Health Addict. (2022) 20:1824–33. doi: 10.1007/s11469-021-00483-5, 33495691 PMC7817067

[10] Borgers N Hox J Sikkel D. Response effects in surveys on children and adolescents: the effect of number of response options, negative wording, and neutral mid-point. Qual Quant. (2004) 38:17–33. doi: 10.1023/B:QUQU.0000013236.29205.a6

[11] Mellor D Moore KA. The use of Likert scales with children. J Pediatr Psychol. (2014) 39:369–79. doi: 10.1093/jpepsy/jst079, 24163438

[12] Ravens-Sieberer U Devine J Bevans K Riley AW Moon J Salsman JM . Subjective well-being measures for children were developed within the PROMIS project: presentation of first results. J Clin Epidemiol. (2014) 67:207–18. doi: 10.1016/j.jclinepi.2013.08.018, 24295987 PMC4120943

[13] Tomyn AJ Fuller-Tyszkiewicz MD Cummins RA Norrish JM. The validity of subjective wellbeing measurement for children: evidence using the personal wellbeing index—school children. J Happiness Stud. (2017) 18:1859–75. doi: 10.1007/s10902-016-9804-3

[14] Casas F Rees G. Measures of children's subjective well-being: analysis of the potential for cross-national comparisons. Child Indic Res. (2015) 8:49–69. doi: 10.1007/s12187-014-9293-z

[15] Cho EY-N Yu F-Y. A review of measurement tools for child wellbeing. Child Youth Serv Rev. (2020) 119:105576. doi: 10.1016/j.childyouth.2020.105576

[16] Levis B Benedetti A Thombs BD DEPRESsion Screening Data (DEPRESSD) Collaboration. Accuracy of patient health Questionnaire-9 (PHQ-9) for screening to detect major depression: individual participant data meta-analysis. BMJ. (2019) 365:l1476. doi: 10.1136/bmj.l1476, 30967483 PMC6454318

[17] Putnick DL Bornstein MH. Measurement invariance conventions and reporting: the state of the art and future directions for psychological research. Dev Rev. (2016) 41:71–90. doi: 10.1016/j.dr.2016.06.004, 27942093 PMC5145197

[18] Sischka PE Martin G Residori C Hammami N Page N Schnohr C . Cross-national validation of the WHO-5 well-being index within adolescent populations: findings from 43 countries [Advance online publication. Assessment. (2025):10731911241309452. doi: 10.1177/10731911241309452 39884717

[19] Fung SF Kong CYW Liu YM Huang Q Xiong Z Jiang Z . Validity and psychometric evaluation of the Chinese version of the 5-item WHO well-being index. Front Public Health. (2022) 10:872436. doi: 10.3389/fpubh.2022.872436, 35433612 PMC9005828

[20] Cosma A Költő A Chzhen Y Kleszczewska D Kalman M Martin G. Measurement invariance of the WHO-5 well-being index: evidence from 15 European countries. Int J Environ Res Public Health. (2022) 19:9798. doi: 10.3390/ijerph19169798, 36011429 PMC9407912

[21] Brown TA. Confirmatory factor analysis for applied research. 2nd ed. New York: Guilford Press (2015).

[22] Chen FF. Sensitivity of goodness of fit indexes to lack of measurement invariance. Struct Equ Modeling. (2007) 14:464–504. doi: 10.1080/10705510701301834

[23] Cheung GW Rensvold RB. Evaluating goodness-of-fit indexes for testing measurement invariance. Struct Equ Modeling Multidiscip J. (2002) 9:233–55. doi: 10.1207/S15328007SEM0902_5

[24] Quansah F Hagan JE Jr Ankomah F Agormedah EK Nugba RM Srem-Sai M . Validation of the WHO-5 well-being scale among adolescents in Ghana: evidence-based assessment of the internal and external structure of the measure. Children. (2022) 9:991. doi: 10.3390/children9070991, 35883975 PMC9323714

[25] Hirota T Adachi M Takahashi M Mori H Shinkawa H Sakamoto Y . Cohort profile: the assessment from preschool to puberty–longitudinal epidemiological (APPLE) study in Hirosaki, Japan. Int J Epidemiol. (2022) 50:1782–1783h. doi: 10.1093/ije/dyab112, 34999860

[26] Adachi M Takahashi M Shinkawa H Mori H Nishimura T Nakamura K. Longitudinal association between smartphone ownership and depression among schoolchildren under COVID-19 pandemic. Soc Psychiatry Psychiatr Epidemiol. (2022) 57:239–43. doi: 10.1007/s00127-021-02196-5, 34773141 PMC8588933

[27] Takahashi M Adachi M Nishimura T Hirota T Yasuda S Kuribayashi M . Prevalence of pathological and maladaptive internet use and the association with depression and health-related quality of life in Japanese elementary and junior high school-aged children. Soc Psychiatry Psychiatr Epidemiol. (2018) 53:1349–59. doi: 10.1007/s00127-018-1605-z, 30255383

[28] Inagaki H Ito K Sakuma N Sugiyama M Okamura T Awata S. Reliability and validity of the simplified Japanese version of the WHO-five well-being index (S-WHO-5-J). Nihon Koshu Eisei Zasshi. (2013) 60:294–301. doi: 10.11236/jph.60.5_294 23942026

[29] Kroenke K Spitzer RL Williams JB. The PHQ-9: validity of a brief depression severity measure. J Gen Intern Med. (2001) 16:606–13. doi: 10.1046/j.1525-1497.2001.016009606.x, 11556941 PMC1495268

[30] Johnson JG Harris ES Spitzer RL Williams JBW. The patient health questionnaire for adolescents: validation of an instrument for the assessment of mental disorders among adolescent primary care patients. J Adolesc Health. (2002) 30:196–204. doi: 10.1016/S1054-139X(01)00333-0, 11869927

[31] Adachi M Takahashi M Hirota T Shinkawa H Mori H Saito T . Distributional patterns of item responses and total scores of the patient health questionnaire for adolescents in a general population sample of adolescents in Japan. Psychiatry Clin Neurosci. (2020) 74:628–9. doi: 10.1111/pcn.13148, 32990411 PMC7702070

[32] Ravens-Sieberer U Erhart M Rajmil L Herdman M Auquier P Bruil J . Reliability, construct and criterion validity of the KIDSCREEN-10 score: a short measure for children and adolescents’ well-being and health-related quality of life. Qual Life Res. (2010) 19:1487–500. doi: 10.1007/s11136-010-9706-5, 20668950 PMC2977059

[33] Nezu S Iwasaka H Saeki K Obayashi K Ishizuka R Goma H . Reliability and validity of Japanese versions of KIDSCREEN-27 and KIDSCREEN-10 questionnaires. Environ Health Prev Med. (2016) 21:154–63. doi: 10.1007/s12199-016-0510-x, 26883049 PMC4823216

[34] Dunn TJ Baguley T Brunsden V. From alpha to omega: a practical solution to the pervasive problem of internal consistency estimation. Br J Psychol. (2014) 105:399–412. doi: 10.1111/bjop.12046, 24844115

[35] Hayes AF Coutts JJ. Use omega rather than Cronbach’s alpha for estimating reliability. But…. Commun Methods Meas. (2020) 14:1–24. doi: 10.1080/19312458.2020.1718629

[36] Tavakol M Dennick R. Making sense of Cronbach’s alpha. Int J Med Educ. (2011) 2:53–5. doi: 10.5116/ijme.4dfb.8dfd, 28029643 PMC4205511

[37] Pfadt JM van den Bergh DVD Sijtsma K Wagenmakers EJ. A tutorial on Bayesian single-test reliability analysis with JASP. Behav Res Methods. (2023) 55:1069–78. doi: 10.3758/s13428-021-01778-0, 35581436 PMC10126026

[38] MacCallum RC Widaman KF Preacher KJ Hong S. Sample size in factor analysis: the role of model error. Multivariate Behav Res. (2001) 36:611–37. doi: 10.1207/S15327906MBR3604_06, 26822184

[39] Worthington RL Whittaker TA. Scale development research: a content analysis and recommendations for best practices. Counsel Psychol. (2006) 34:806–38. doi: 10.1177/0011000006288127

[40] Browne MW. An overview of analytic rotation in exploratory factor analysis. Multivar Behav Res. (2001) 36:111–50. doi: 10.1207/S15327906MBR3601_05

[41] Kaiser HF. The application of electronic computers to factor analysis. Educ Psychol Meas. (1960) 20:141–51. doi: 10.1177/001316446002000116

[42] Cattell RB. The scree test for the number of factors. Multivariate Behav Res. (1966) 1:245–76. doi: 10.1207/s15327906mbr0102_10, 26828106

[43] Hu LT Bentler PM. Cutoff criteria for fit indexes in covariance structure analysis: conventional criteria versus new alternatives. Struct Equat Model Multidiscipl J. (1999) 6:1–55. doi: 10.1080/10705519909540118

[44] Byrne BM. Structural equation modeling with AMOS: basic concepts, applications, and programming. 2nd ed. New York: Routledge/Taylor & Francis Group (2010).

[45] Kenny DA Kaniskan B McCoach DB. The performance of RMSEA in models with small degrees of freedom. Sociol Methods Res. (2015) 44:486–507. doi: 10.1177/0049124114543236

[46] Hosmer DW Lemeshow S Sturdivant RX. Applied logistic regression. 3rd ed. New York: John Wiley & Sons (2013).

[47] Youden WJ. Index for rating diagnostic tests. Cancer. (1950) 3:32–5. doi: 10.1002/1097-0142(1950)3:1<32::AID-CNCR2820030106>3.0.CO;2-3, 15405679

[48] Deeks JJ Altman DG. Diagnostic tests 4: likelihood ratios. BMJ. (2004) 329:168–9. doi: 10.1136/bmj.329.7458.168, 15258077 PMC478236

[49] Jaeschke R Guyatt GH Sackett DL Guyatt G Bass E Brill-Edwards P . Users' guides to the medical literature: III. How to use an article about a diagnostic test B. What are the results and will they help me in caring for my patients? JAMA. (1994) 271:703–7. doi: 10.1001/jama.1994.03510330081039 8309035

[50] Kim H-Y. Statistical notes for clinical researchers: assessing normal distribution (2) using skewness and kurtosis. Restor Dent Endod. (2013) 38:52–4. doi: 10.5395/rde.2013.38.1.52, 23495371 PMC3591587

[51] Kline RB. Principles and practice of structural equation modeling. 4th ed. New York: Guilford Press (2016).

[52] Cohen J. Statistical Power analysis for the behavioral science. 2nd ed. Hillsdale, NJ: Lawrence Erlbaum Associates (1988) doi: 10.4324/9780203771587.

[53] Hanley JA McNeil BJ. The meaning and use of the area under a receiver operating characteristic (ROC) curve. Radiology. (1982) 143:29–36. doi: 10.1148/radiology.143.1.7063747, 7063747

[54] Brehaut E Neupane D Levis B Wu Y Sun Y Ioannidis JPA . ‘Optimal’ cutoff selection in studies of depression screening tool accuracy using the PHQ-9, EPDS, or HADS-D: a meta research study. Int J Methods Psychiatr Res. (2023) 32:e1956. doi: 10.1002/mpr.1956, 36461893 PMC10485315

[55] Löwe B Spitzer RL Gräfe K Kroenke K Quenter A Zipfel S . Comparative validity of three screening questionnaires for DSM-IV depressive disorders and physicians’ diagnoses. J Affect Disord. (2004) 78:131–40. doi: 10.1016/S0165-0327(02)00237-9, 14706723

[56] Spitzer RL Kroenke K Williams JBW Patient Health Questionnaire Primary Care Study Group. Validation and utility of a self-report version of PRIME-MD: the PHQ primary care study. Primary care evaluation of mental disorders. Patient health questionnaire. JAMA. (1999) 282:1737–44. doi: 10.1001/jama.282.18.1737, 10568646

[57] Streiner DL Norman GR Cairney J. Health measurement scales: a practical guide to their development and use. 5th ed. New York: Oxford University Press (2015).

[58] Sijtsma K. On the use, the misuse, and the very limited usefulness of Cronbach’s alpha. Psychometrika. (2009) 74:107–20. doi: 10.1007/s11336-008-9101-0, 20037639 PMC2792363

[59] Halliday JA Hendrieckx C Busija L Browne JL Nefs G Pouwer F . Validation of the WHO-5 as a first-step screening instrument for depression in adults with diabetes: results from diabetes MILES – Australia. Diabetes Res Clin Pract. (2017) 132:27–35. doi: 10.1016/j.diabres.2017.07.005, 28783530

[60] Habibzadeh F Habibzadeh P Yadollahie M. On determining the most appropriate test cut-off value: the case of tests with continuous results. Biochem Med. (2016) 26:297–307. doi: 10.11613/BM.2016.034, 27812299 PMC5082211

[61] Trevethan R. Sensitivity, specificity, and predictive values: foundations, pliabilities, and pitfalls in research and practice. Front Public Health. (2017) 5:307. doi: 10.3389/fpubh.2017.00307, 29209603 PMC5701930

[62] Zimmerman M. Positive predictive value: a clinician’s guide to avoid misinterpreting the results of screening tests. J Clin Psychiatry. (2022) 83:22com14513. doi: 10.4088/JCP.22com14513, 36005892

[63] World Health Organization (2021). Mental health of adolescents. Available online at: https://www.who.int/news-room/fact-sheets/detail/adolescent-mental-health

[64] Polanczyk GV Salum GA Sugaya LS Caye A Rohde LA. Annual research review: a meta-analysis of the worldwide prevalence of mental disorders in children and adolescents. J Child Psychol Psychiatry. (2015) 56:345–65. doi: 10.1111/jcpp.12381, 25649325

[65] Avenevoli S Swendsen J He JP Burstein M Merikangas KR. Major depression in the national comorbidity survey-adolescent supplement: prevalence, correlates, and treatment. J Am Acad Child Adolesc Psychiatry. (2015) 54:37–44.e2. doi: 10.1016/j.jaac.2014.10.010, 25524788 PMC4408277

[66] Andermann A Blancquaert I Beauchamp S Déry V. Revisiting Wilson and Jungner in the genomic age: a review of screening criteria over the past 40 years. Bull World Health Organ. (2008) 86:317–9. doi: 10.2471/BLT.07.050112, 18438522 PMC2647421

[67] Wilson J.M.G. Jungner G. (1968) “Principles and practice of screening for disease,” Public health papers no. 34 Geneva World Health Organization. Available online at: https://apps.who.int/iris/handle/10665/37650

[68] Kagee A Tsai AC Lund C Tomlinson M. Screening for common mental disorders in low resource settings: reasons for caution and a way forward. Int Health. (2013) 5:11–4. doi: 10.1093/inthealth/ihs004, 23580905 PMC3619733

[69] Karcher NR Hicks R Schiffman J Asarnow JR Calkins ME Dauberman JL . Youth mental health screening and linkage to care. Psychiatr Serv. (2023) 74:727–36. doi: 10.1176/appi.ps.202200008, 36695011 PMC10329990

[70] Young JF Miller MR Khan N. Screening and managing depression in adolescents. Adolesc Health Med Ther. (2010) 1:87–95. doi: 10.2147/AHMT.S7539, 24600264 PMC3916013

[71] Allgaier AK Pietsch K Frühe B Sigl-Glöckner J Schulte-Körne G. Screening for depression in adolescents: validity of the patient health questionnaire in pediatric care. Depress Anxiety. (2012) 29:906–13. doi: 10.1002/da.21971, 22753313

