# Supplementary material for: Psychometric validation of the WHO-5 and WHO-4 well-being index scales for assessing psychological well-being and detecting depression in Japanese school-aged children: a community-based study
Source: Front Public Health. 2025 Dec 10;13:1662332. doi: 10.3389/fpubh.2025.1662332 (PMC12727617; doi:10.3389/fpubh.2025.1662332)
Supplement: Supplementary file 1 [file Supplementary_file_1.pdf]

frontiers

Supplementary Material

1. Supplementary Text. WHO-5-J (Child Version): Translation and Cultural / Developmental Adaptation Procedures

Basis and scope. We used the S-WHO-5-J—a reliability- and validity-verified instrument for Japanese adults and older adults—as a reference, but the child version was primarily anchored to the official WHO-5 source text issued by the World Health Organization (WHO, 2024; CC BY-NC-SA 3.0 IGO). The aim was to produce wording that typical pupils in Grades 4–9 could read and answer accurately without adult mediation.

Multidisciplinary rewriting team. A team of three nationally licensed teachers (holders of Japanese national teaching certificates, including specialists in elementary education and special needs education) and three nationally licensed psychologists (Licensed Psychologists, specializing in child/adolescent practice) jointly rewrote the instructions and all item statements to make them concrete, concise, and age-appropriate.
Focused review of Item 1 and related wording. Because WHO-5 Item 1 has been debated internationally with respect to cross-cultural measurement invariance, we conducted a targeted item-level review. In Japanese, the English nuances of *cheerful*, *active*, and *vigorous* often collapse into the everyday word "genki." To avoid loss of meaning and to maintain item differentiation, the research board met repeatedly and adopted distinct, child-understandable phrasings that preserved the intended constructs, for example:

• *cheerful* → "feeling bright and in a good mood" (hedonic affect, not energy)
• *active* → "being able to do things actively" (behavioral activation)
• *vigorous* → "feeling full of energy" (high arousal/energy)

These choices were retained only when they preserved the semantic and conceptual content of the source items.

Step-by-step translation assurance.

1. Forward translation and consensus. The child-adapted Japanese draft was produced within the team and finalized by consensus after a line-by-line review against the WHO source text.
2. Single Back Translation. An independent translator, blinded to the original English, translated the Japanese draft back into English; a separate reviewer compared the original and back-translated English to identify meaning drift and resolve discrepancies.
3. Level-3 native check. Under the Level-3 process, a native-language specialist conducted a final native check after back translation to ensure idiomatic clarity and consistency.

2. Supplementary Tables S1–S2. Age-stratified diagnostic accuracy of WHO-4 and WHO-5 for detecting depression severity (Elementary and Junior-high strata)

Supplementary Table S1. Diagnostic accuracy of the WHO-4 and WHO-5 for detecting depression severity (Elementary; Grades 4–6)

Dependent Variable (Severity of Depression)	Predictor	AUC	SE	Asymptotic p-value	95% CI		N (%)	
					Lower	Upper	Positive	Negative
Moderate depression	WHO-4	0.83	0.01	p < .001	0.81	0.85	384 (11.5)	2962 (88.5)
	WHO-5	0.83	0.01	p < .001	0.81	0.86		
Moderately severe depression	WHO-4	0.86	0.02	p < .001	0.82	0.9	125 (3.7)	3221 (96.3)
	WHO-5	0.87	0.02	p < .001	0.83	0.9		
Severe depression	WHO-4	0.91	0.03	p < .001	0.85	0.97	37 (1.1)	3309 (98.9)
	WHO-5	0.91	0.03	p < .001	0.85	0.97		

Supplementary Table S2. Diagnostic accuracy of the WHO-4 and WHO-5 for detecting depression severity (Junior-high; Grades 7–9)

Dependent Variable (Severity of Depression)	Predictor	AUC	SE	Asymptotic p-value	95% CI		N (%)	
					Lower	Upper	Positive	Negative
Moderate depression	WHO-4	0.86	0.01	p < .001	0.84	0.88	388 (11.6)	2947 (88.4)
	WHO-5	0.86	0.01	p < .001	0.84	0.88		
Moderately severe depression	WHO-4	0.91	0.01	p < .001	0.88	0.93	134 (4.0)	3201 (96.0)
	WHO-5	0.91	0.01	p < .001	0.88	0.93		
Severe depression	WHO-4	0.93	0.02	p < .001	0.88	0.97	38 (1.1)	3297 (98.9)
	WHO-5	0.93	0.02	p < .001	0.88	0.97		

Note. WHO-5 = 5-item World Health Organization Well-Being Index; WHO-4 = modified 4-item version excluding Item 1; AUC = area under the curve; SE = standard error; CI = confidence interval; N (%) = number and percentage of participants classified by PHQ-A–based

severity cutoffs (≥10 for moderate, ≥15 for moderately severe, ≥20 for severe). All analyses were statistically significant at $p < .001$. Higher AUC values indicate better diagnostic accuracy.
